# Towards better delineation of hydrothermal alterations via multi-sensor remote sensing and airborne geophysical data

**DOI:** 10.1038/s41598-023-34531-y

**Published:** 2023-05-06

**Authors:** Ali Shebl, Mahmoud Abdellatif, Mohamed Badawi, Maher Dawoud, Amr S. Fahil, Árpád Csámer

**Affiliations:** 1 Department of Mineralogy and Geology, University of Debrecen, Debrecen, 4032 Hungary; 2 Department of Geology, Tanta University, Tanta, 31527 Egypt; 3 Department of Geology, South Valley University, Qena, 83523 Egypt; 4 Institute of Exploration Geosciences, University of Miskolc, Miskolc, 3515 Hungary; 5 Department of Geology, Mansoura University, Mansoura, 35516 Egypt; 6 Department of Geology, Faculty of Science, Menoufia University, Shibin El-koom, Egypt; 7 Department of Earth and Ocean Sciences, University of North Carolina Wilmington, 601 South College Road, Wilmington, NC 28403-5944 USA

**Keywords:** Geophysics, Economic geology

## Abstract

Integrating various tools in targeting mineral deposits increases the chance of adequate detection and characterization of mineralization zones. Selecting a convenient dataset is a key for a precise geological and hydrothermal alteration mapping. Remote sensing and airborne geophysical data have proven their efficiency as tools for reliable mineral exploration. Advanced Spaceborne Thermal Emission and Reflection Radiometer (ASTER), Advanced land imager (ALI), Landsat 8 (L8), and Sentinel 2 data are widely-used data among various types of remote sensing images in resolving lithological and hydrothermal alteration mapping over the last two decades. ASTER is a well-established satellite in geological remote sensing with detailed Short-wave infrared (SWIR) range compared to visible and near-infrared region (VNIR) that controls iron-associated alteration detection. On contrary, ALI has excellent coverage of the VNIR area (6 bands), but does not possess the potentiality of ASTER for the SWIR and thermal regions. Landsat 8 is widely used and highly recommended for lithological and hydrothermal alteration mapping. The higher spatial (up to 10 m) resolution of Sentinel 2 MSI has preserved its role in producing accurate geological mapping. Notwithstanding the foregoing, implementing the four datasets in a single study is time-consuming. Thus, an important question when commencing an exploration project for hydrothermal alterations-related mineralization (orogenic mineral deposits in the current research) is: which dataset should be adopted to fulfill proper and adequate outputs? Here the four widely recommended datasets (ASTER, ALI, L8, and sentinel 2) have been tested by applying the widely-accepted techniques (false color combinations, band ratios, directed principal component analysis, and constrained energy minimization) for geological and hydrothermal alteration mapping of Gabal El Rukham-Gabal Mueilha district, Egypt. The study area is covered mainly by Neoproterozoic heterogeneous collection of ophiolitic components, island arc assemblage, intruded by enormous granitic rocks. Additionally, airborne magnetic and radiometric data were applied and compared with the remote sensing investigations for deciphering the structural and hydrothermal alteration patterns within the study area. The results demonstrated a different extent from one sensor to another, highlighting their varied efficacy in detecting hydrothermal alterations (mainly hydroxyl-bearing alterations and iron oxides). Moreover, the analysis of airborne magnetic and radiometric data showed hydrothermal alteration zones that are consistent with the detected alteration pattern. The coincidence between high magnetic anomalies, high values of the K/eTh ratio, and the resultant alterations confirm the real alteration anomalies. Over and above that, the remote sensing results and airborne geophysical indications were verified with fieldwork and petrographic investigations, and strongly recommend combining ASTER and Sentinel 2 results in further investigations. Based on the outputs of the current research, we expect better hydrothermal alteration delineation by adopting the current findings as they sharply narrow the zones to be further investigated via costly geophysical and geochemical methods in mineral exploration projects.

## Introduction

The use of remote sensing in the exploration of mineral deposits has been widely employed^1–3^. The fundamental key in these studies is the ability of remote sensing datasets to detect the associated hydrothermal alteration zones. These zones could even be distinguished according to the detected mineral(s) that mostly manifest unique spectral characteristics in VNIR and SWIR regions^4,5^. For instance, in SWIR, a sharp Al–OH absorption trough closer to 2200 nm together with another minor absorption feature approximately at 2350 nm indicate the presence of muscovite that could be a guide for phyllic alteration (mostly muscovite and sericite)^6^. Argillic alteration, particularly kaolinite, could be recognized through a major absorption feature at 2200 nm with a minor one at 2170 nm. Outer zones of propylitic alteration are commonly identified by the presence of chlorite and epidote. The latter is distinguished by a deep Mg–OH absorption trough, besides a shallow one at 2250 nm^6^. In VNIR, limonite, for example, exhibits a strong absorption feature at 480 nm and a secondary trough at 930 nm. However, detection of these minerals does not directly indicate the presence of economic mineral deposits (i.e., feldspar weathering mostly results in kaolinite and epidote formation, which may also be attributed to regional metamorphism). Thus, a systematic geological interpretation is a must, and, in most cases, higher interrelated muscovite, epidote, and kaolinite anomalies are considered a piece of evidence for mineral deposits^7^.

The identification of minerals is strongly linked to the spectral range used, and therefore, the choice of sensor is primarily determined by the spectral range it covers (which is the main criterion for selecting an appropriate sensor for a particular application)^8^. Recently, the identification of high-potential mineralized zones has received significant interest in the geological community due to its positive indications in outlining significant mineral resources. Several studies have applied different approaches including band ratios^1,2,9,10^, selective principal component analysis (SPCA)^11,12^, mixture-tuned matched-filtering^13^, dirichlet process based on stick-breaking model-based clustering^14^, evidential belief functions^15^, neuro-fuzzy-AHP^16^, fuzzy logic^17,18^, and multi-class index overlap^17^, over various remote-sensing datasets as a low-cost tool for mineral exploration. Among these various datasets and for the purpose of geological mapping, researchers widely utilized four main optical sensors. ASTER, with its powerful six SWIR bands, was and is still a pioneer satellite in geological applications^4,7,12,19–22^. ASTER’s detailed SWIR coverage allows the recognition of several hydrothermal alteration minerals, including illite*/*muscovite (sericite), kaolinite, epidote, calcite, and chlorite, due to distinctive absorption features resulting from Al–OH, Mg–OH, H–O–H, and CO_3_^−^. The Advanced Land Imager (ALI) is preferred for providing detailed information on the absorption characteristics of transition metals (such as iron) because it has six VNIR bands, whereas ASTER only has three^23–27^. Multispectral Scanner Landsat data was the first dataset utilized in the mapping of iron oxides in the 1980s^28^. Then, Thematic Mapper (TM) Landsat spectral bands were useful to distinguish ferric/ferrous oxides (b3/b1) and argillic/non-argillic compositions (b5/b7) by utilizing the band ratio technique. As a continuation of TM and ETM Landsat data, Landsat 8 OLI (Operational Land Imager) was extensively used in geological mapping^10,29–34^. Recently, Sentinel 2, provided by the European Space Agency (ESA), has evidenced its power in geological and hydrothermal alteration mapping. For instance, ferric iron, ferrous iron, laterite, gossan, ferrous silicate, and ferric oxides, besides other types of alteration are recognized^7,12,33,35,36^.

Irrespective of the utilized sensor and its capability, remote sensing data should be integrated with geophysical and/or geochemical data for better delineation of hydrothermal alterations-related mineralizations and result verification (through analyzing the harmony and matching among multisource datasets). Airborne geophysical data (e.g., magnetic, radiometric) has proven its efficiency and capability in detecting hydrothermal alteration tracts associated with mineral deposits. Aeromagnetic data allows delineating structural patterns that serve as channels for hydrothermal alteration and/or detecting altered zones connected to porphyry minerals^37–39^. Also, the aeroradiometric data via arithmetic combination (the ratio of two radioelement grids), for example, the ratio of potassium counts and equivalent thorium concentrations (K/eTh) or its inverted form^40,41^, the F-parameter (the ratio of the product of potassium (K) counts and equivalent uranium (eU) concentration to the equivalent of thorium (eTh) concentration) suggested by Efimov^42^, and Kd the deviation from the potassium (K) counts^43^ are highly recommended for mapping potassic altered regions.

Consequently, one of the main targets of the current research is resolving the hydrothermal alteration patterns and structural framework of the study area based on multiscale (satellite remote sensing images, airborne geophysical data, fieldwork, and petrographic investigations) and multisource (multi-sensor remote sensing images with different spectral and spatial characteristics, magnetic, and radiometric grids) data to increase the probability of locating real alteration zones for further feasibility studies and exploitation. Additionally, the current research is an attempt to highlight a suitable dataset that well-matches ASTER findings in hydrothermal alteration mapping (as ASTER encounters some issues especially related to the availability of SWIR bands) through a comparative study performed with the widely utilized sensors for better identification of alteration zones. As using the four sensors to decipher the lithological and hydrothermal regimes of a particular study is time-consuming. We seek to define the true hydrothermal anomalies and assess their suitability and magnitude. Toward our aim, the well-known techniques, including false color combination (FCC), band ratio (BR), relative absorption band depth (RBD), directed principal component analysis (DPCA), and constrained energy minimization (CEM), are utilized. Additionally, our outputs are verified with aeromagnetic data (delineating the structural complexity), spectrometric gamma-ray data (highlighting the alteration zones), field observations, and petrographic investigations.

## Study area and geological setting

**Figure 1:**
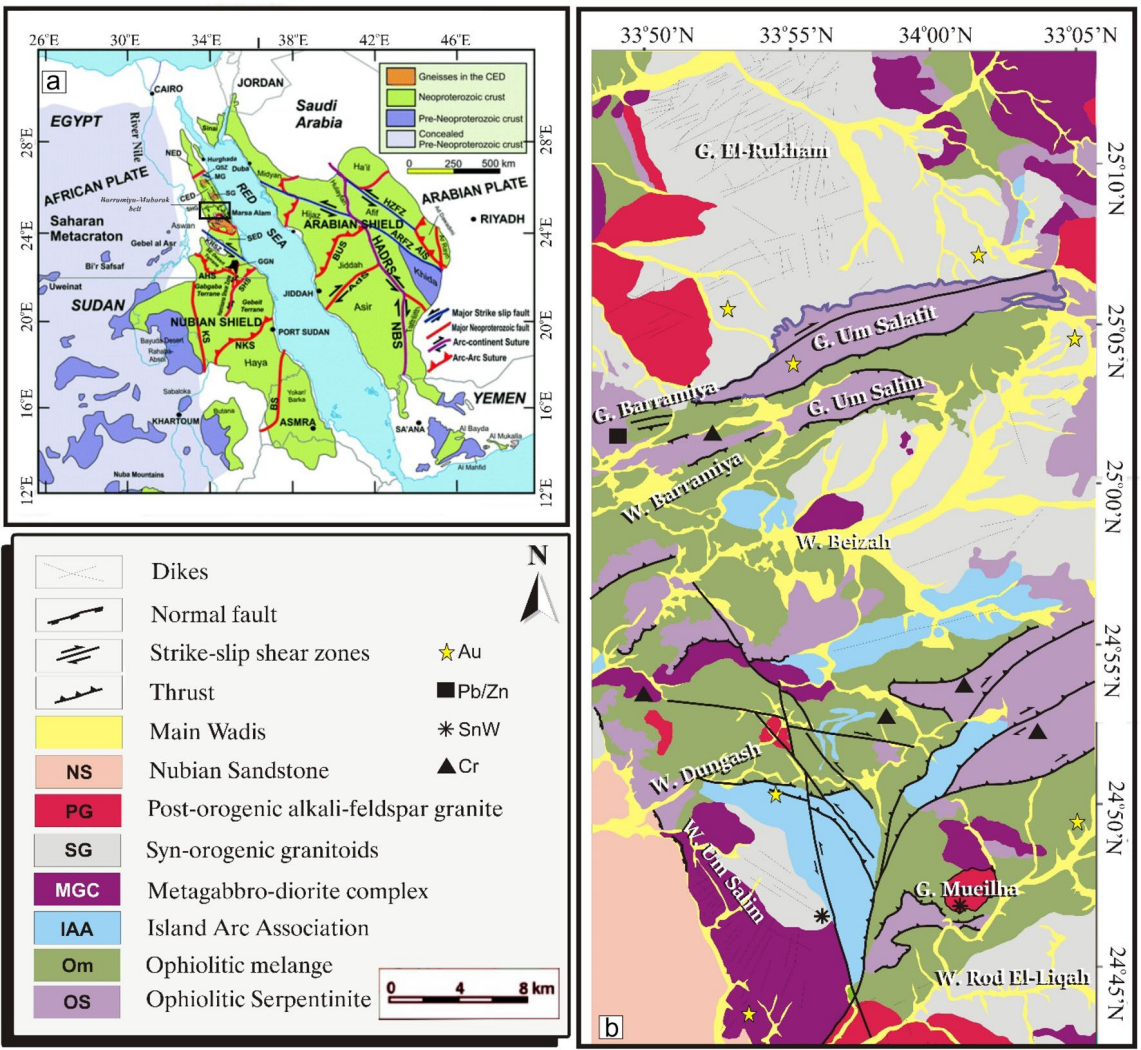
(**a**) A simplified geological map of the Arabian Nubian Shield modified after Johnson et al.^45^ showing the location of the Barramiya-Mubarak belt (small black rectangle) where the study area is located, and (**b**) a geological map of the study area showing its lithological units, modified after Zoheir et al.^49^. (Created by SmartSketch v. 4.0 software; https://smartsketch.software.informer.com/4.0/).

The area of study includes Gabal El Rukham-Gabal Mueilha district, Eastern Desert, Egypt. They constitute the northwestern part of the Nubian Shield (Fig. 1a). This shield was developed through a prolonged series of orogenic events, referred to the Pan African Orogeny (~ 950 to 550 Ma^44,45^), manifesting a superimposed tectonic framework of ductile and brittle deformations (D1, D2, and D3^46,47^). This orogeny was accompanied by Neoproterozoic Pan-African thrusting (e.g., Beitan, Hafafit, Barramiya, El-Shalul, Meatiq), that is separated as suprastructural units from infrastructural gneisses, migmatites, and cleaved granites. Except for the southwest corner, covered by the Phanerozoic Nubian Sandstone, the majority of the study area is occupied by ophiolitic serpentinite, metagabbro, ophiolitic mélange, metavolcanics, and syn- to post-tectonic granite (Fig. 1b). This regime, which is characterized by diverse lithologies derived from various tectonic protoliths, is commonly associated with a number of mineral deposits (e.g., Au, Cu, and Sn) reflecting remarkable structural control and association with hydrothermal zones. The study area possesses various lithologies and favorable conditions for hydrothermal alterations and the associated mineralizations. In addition, it contains fifteen metallogenic spots^48^ (Fig. 1b) that could be used as spatial verification points (besides the other datasets and field observations) for the results of hydrothermal alteration in the current study. Therefore, the area under consideration provides an excellent case study to evaluate the capability of ASTER, Landsat 8, Sentinel 2, and ALI for lithologic mapping utilizing the widely used FCCs and PCA in conjunction with the previous geological maps^24,49,50^.

Moreover, the ANS has structural and economic significance due to the presence of valuable base metals (e.g., gold, copper, zinc, silver, sulfides, chromite, nickel, etc.^44,45,51–54^). These precious metals, particularly gold deposits are linked with alteration zones in the central and southern Eastern Desert of Egypt (where the study area is located) developed by hydrothermal activities, induced by metamorphic or hydrothermal off-sets of unexposed intrusions (e.g., Barrmiya and Dungash^49,55–57^). Several types of alteration zones are recorded in the Barramiya-Dungash region, including listvenite, sericitization, sulphidization, carbonatization, and chloritization. In the Barrmiya area, gold mineralization is associated with listvenite, which developed along the sheared serpentinites and close to chromite, magnesite, and antimony ores^58,59^. While in the Dungash area, gold mineralization is associated with alteration zones of varying mineralogy superimposing a metamorphic assemblage of greenschist facies^56^.

## Materials and methods

### Remote sensing data characteristics and pre-processing

In this study, the ASTER, ALI, L8, and Sentinel 2 datasets are used to map lithological units and hydrothermal alterations. Table 1 shows the spectral and spatial characteristics of each sensor. ASTER was launched on the Terra platform in December 1999. In the current study, ASTER scene was obtained from the USGS (https://earthexplorer.usgs.gov/). Earth Observing-1 (EO-1) started its mission on November 21, 2000, to provide multispectral data using ALI and hyperspectral data through Hyperion. For the current investigation, ALI data with a spatial resolution of 30 m for the multispectral bands and 10 m for the panchromatic band is applied. The Landsat 8 satellite was launched on February 11, 2013, and was equipped with two sensors, the Operational Land Imager (OLI) and the Thermal Infrared Sensor (TIRS), which allowed it to acquire high radiometric resolution (16-bit) data in the VNIR, SWIR, and TIR regions, as shown in Table 1. For the current purpose, a level 1 T (terrain corrected) scene was utilized. The Sentinel-2 satellite was developed by the ESA as part of the Copernicus Programme to provide spectral data in 13 bands with variable spatial resolutions (10, 20, or 60 m). For the current study, Sentinel 2A MSI, as an L1C product data, is accessed through the Copernicus Open Access Hub.

**Table 1:** Characteristics of the utilized optical datasets.

Landsat 8		ASTER	Sentinel 2	EO1 ALI							
Revisit time	16 days	16 days	5 days	16 days							
Swath width	185 km	60 km	290 km	37 km							
Radiometric R	12-bit	8-bit (VNIR-SWIR)	12-bit	12-bit							

Spectral and spatial characteristics											
B.n	C.W. (µm)	S.R (m)	B.n	C.W. (µm)	S.R (m)	B.n	C.W. (µm)	S.R (m)	B.n	C.W. (µm)	S.R (m)
1	0.442	30	1	0.560	15	1	0.443	60	PAN	0.585	10
2	0.483	30	2	0.660	15	2	0.490	10	1	0.443	30
3	0.561	30	3N	0.820	15	3	0.560	10	2	0.482	30
4	0.654	30	3B	0.820	15	4	0.665	10	3	0.565	30
5	0.864	30	4	1.650	30	5	0.704	20	4	0.660	30
6	1.609	30	5	2.165	30	6	0.740	20	5	0.790	30
7	2.203	30	6	2.205	30	7	0.782	20	6	0.867	30
8	0.598	15	7	2.260	30	8	0.842	10	7	1.250	30
9	1.373	30	8	2.330	30	8a	0.865	20	8	1.650	30
10	10.90	100	9	2.395	30	9	0.945	60	9	2.215	30
11	12.00	100				10	1.375	60			
						11	1.610	20			
						12	2.190	20			

Band number (B.n), Central wavelength (C.W), Spatial Resolution (S.R) and PAN for panchromatic.

**Figure 2:**
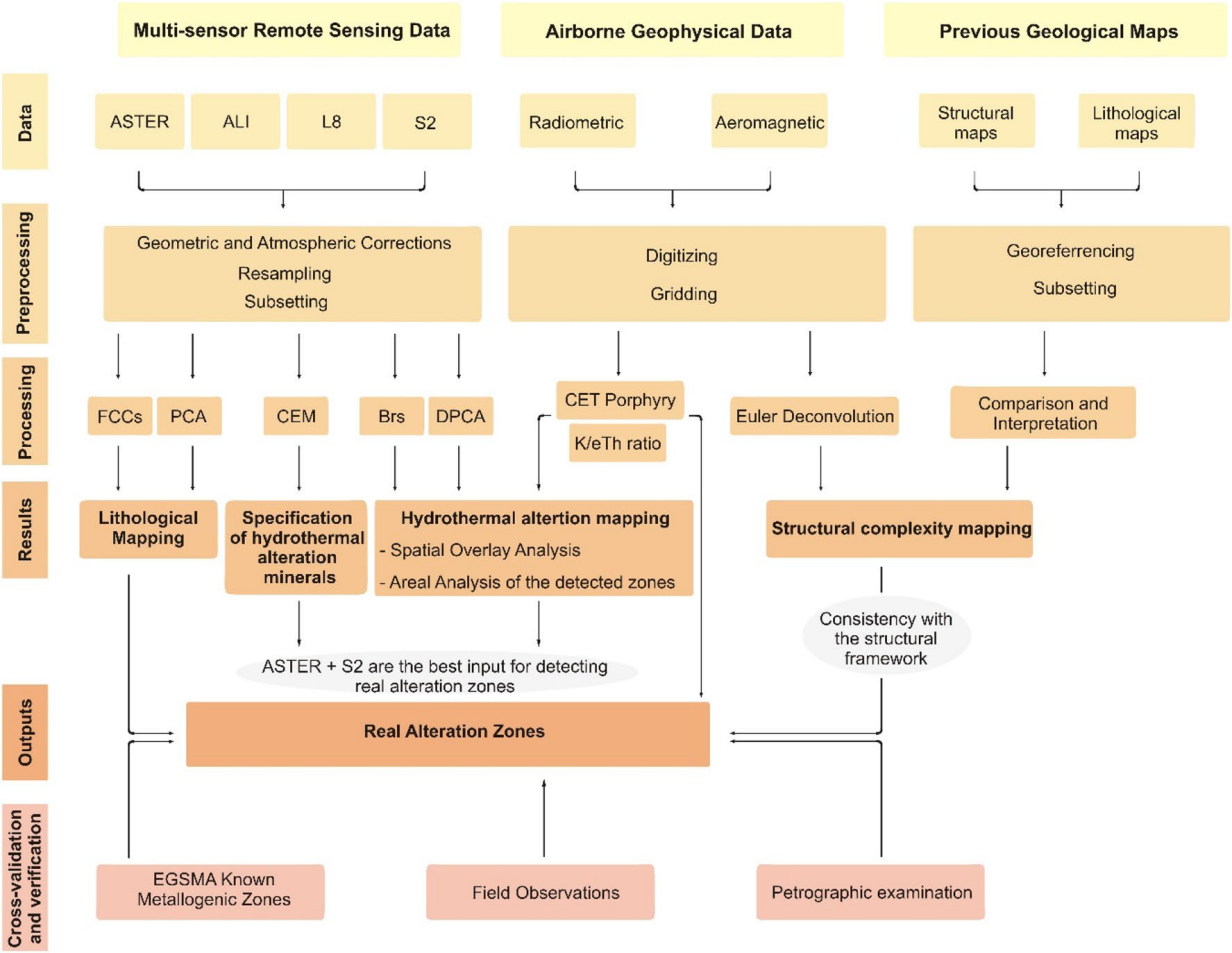
Flowchart showing the adopted methodology utilized in the current study.

Unifying the data characteristics is recommended for such studies as the results may be affected by season (mainly due to the effect of rainfall), time of acquisition (that controls the shadows), and differences in pre-processing. In the current research, four cloud-free scenes were obtained beyond the range of flash flood events (After revising the timings of major flash flood events within the study area and its environs), with the following times of acquisition 07:59:31, 08:03:44, 08:13:29, and 8:16:11 for ALI (EO1A1740422003070110PZ), ASTER (AST_L1A_00303062007083043), Landsat OLI (LC81740432019298LGN00), and Sentinel 2 (S2A_MSIL1C_20200505T081611), respectively. ASTER, ALI, and L8 are preprocessed (geometrically corrected according to UTM, WGS 84 zone 36 N, running Fast Line-of-Sight Atmospheric Analysis of Spectral Hypercubes atmospheric correction) and clipped to the borders of the investigated area. Sentinel 2 data were georeferenced to the zone 36 North UTM projection using the WGS-84 datum, then atmospherically corrected using sen2cor processor in the Sentinel Application Platform (SNAP). All the datasets are resampled (nearest neighbor method) to a 20 m pixel size to allow comparison. A flowchart showing the adopted methodology utilized in the current study is introduced in Fig. 2.

### Image processing methods

**Figure 3:**
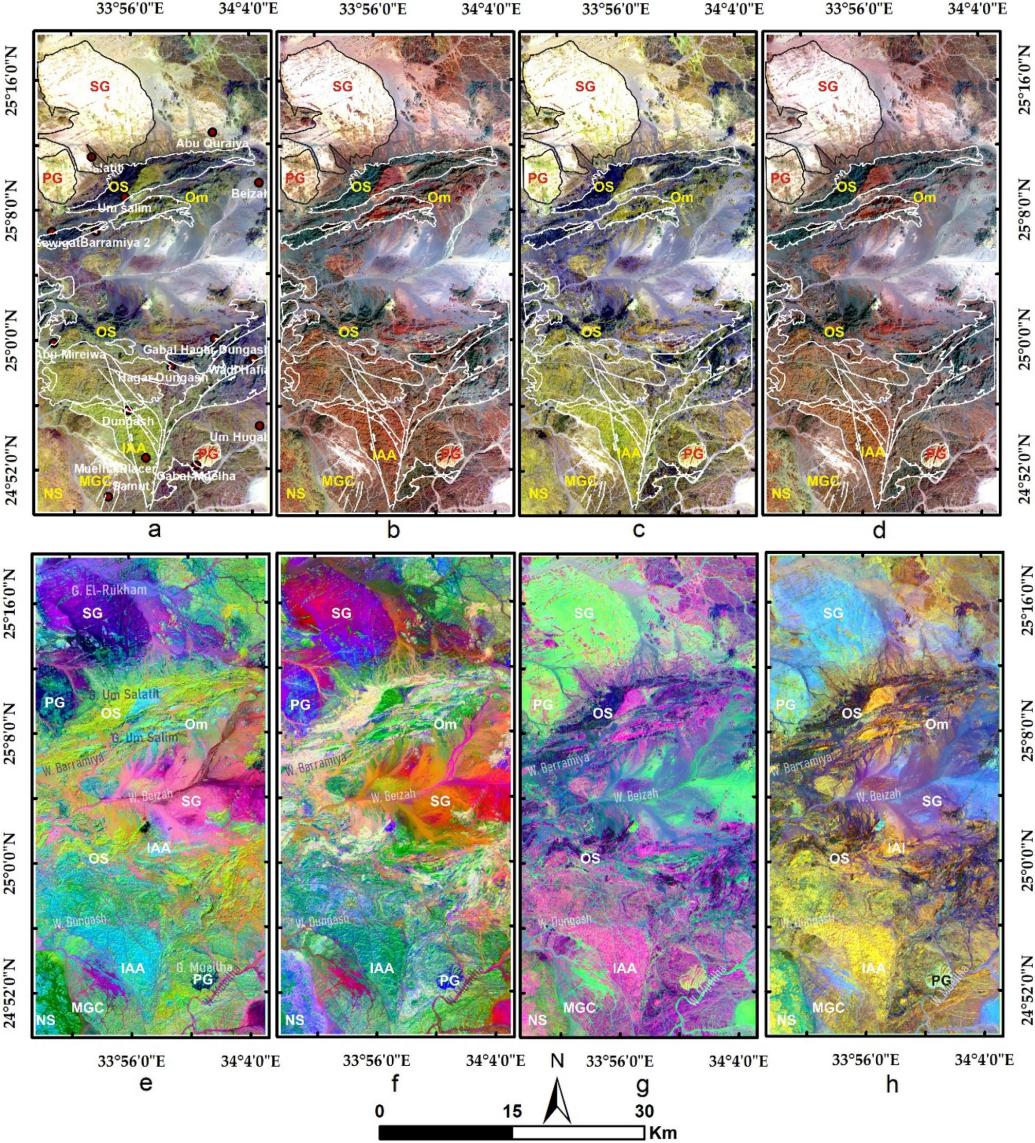
Lithological mapping by RGB FCCs of (**a**) ASTER 9/4/1 with distribution of the metallogenic spots within the study area, (**b**) Landsat OLI 7/5/3, (**c**) Sentinel 2 12/11/2, (**d**) ALI 9/6/3, and PCs of (**e**) ASTER 2/1/3, (f) L8 2/1/3, (**g**) S2 2/1/3, and (**h**) ALI 3/2/1. Abbreviations are given in the geological map (Fig. 1), for instance SG: syn tectonic granite, IAA: island arc association,…etc.

The current study utilized false-color composites (FCCs) and principal component analysis (PCA) for discriminating the different rock units and deciphering the alteration pattern within the study area. According to the data specifications, we focused on discriminating the geological units and comparing the results with each other through overlay analysis and visual comparison. Moreover, the results of the four datasets are compared to each other and correlated with a recently published geologic map of the study area^49^, and the known fifteen metallogenic spots (Figs. 1b and 3a)^48^. Then band ratio (BR), relative absorption band depth (RBD), directed principal component analysis (DPCA), and constrained energy minimization (CEM) techniques have been applied to extract various types of alteration. These methods are adopted in the current research as they are widely accepted in the geological scientific community and their results are reliable according to several previous studies in different terrains^12,32,36,39,60–62^.

#### Band ratio and relative absorption band depth

The Band ratio method is a powerful technique for discriminating different rock and mineral groups through boosting spectral variances and lowering topographical effects^63,64^. For iron-related alterations (sensed via the VNIR spectral range), ALI can detect all iron oxides by two different band ratios, while L8, ASTER and Sentinel 2 can detect them by a single ratio. The four datasets can map ferrous iron, ferric iron, ferric and ferrous oxides, laterite, gossan, ferrous silicates, as shown in Table 2. To configure the whole alteration pattern within the study area, the well-known Sabins ratio^65^ FCC is applied to the four datasets to compare the results with the known altered spots. In this ratio and mimicking Landsat TM results, hydroxyl-bearing alteration minerals are displayed in red, iron oxides (ferric iron in the case of ASTER) are shown in green, and ferrous oxides are represented by bluish colors. These anomalies are sliced and separated after specifying four threshold values related to each ratio statistic and according to the following equation: Th = μ + σ where μ is the mean value and σ represents the standard deviation.

**Table 2:** Band ratios for highlighting various mineral features using the four utilized sensors.

Highlighted mineral feature	ASTER	Landsat 8	Sentinel 2	EO1 ALI
Ferrous iron	5/3 + 1/2	7/5 + 3/4	12/8 + 3/4	9/6 or 9/5 3/4
	(van der Meer et al.^75^; Van der Meer et al.^35^)			
Ferric iron (Fe3+/Fe2+ iron oxides)	2/1	4/3	4/3	4/3
	(Pour et al.^32^; van der Meer et al.^75^; Van der Meer et al.^35^)			
Ferric oxide	4/3	6/5	11/8	8/6 or 8/5
	(Pour et al.^32^; van der Meer et al.^75^; Van der Meer et al.^35^)			
Gossan, (Fe3+/Fe2+ iron oxides) ferrous iron oxides	4/2	6/4	11/4	8/4
	(Pour et al.^32^; van der Meer et al.^75^)			
Laterite	4/5		11/12	
	(Van der Meer et al.^35^)			
Ferrous silicates (Biotite, chloride, amphibole)	5/4		12/11	
	(Van der Meer et al.^35^)			
Ferrous iron oxides	2/4	4/6	4/11	4/8
	(Pour et al.^32^)			
All iron oxides (Fe3+/Fe2+ and Fe–OH iron oxides)	–	4/2	4/2	4/2 or 4/1
	(Pour et al.^32^)			
Hydroxyl bearing alteration (Al–OH and Fe, Mg–OH)	4/5 or 6 or 7	6/7	11/12	8/9
	(Pour et al.^32^)			
Sabins ratio in RGB	4/7, 2/1, 4/3	6/7, 4/2, 6/5	11/12, 4/2, 11/8	8/9, 4/1, 8/6
	(Frutuoso et al. 2021)			
Carbonate/chlorite/epidote Mg–Fe–OH	(7 + 9)/8	ASTER bands 5:7 are only covered by band 7	ASTER bands 5:7 are only covered by band 12	ASTER bands 5:7 are only covered by band 9
	(Ninomiya et al. 2005; Pour et al.^32^; van der Meer et al.^75^)			
Dolomite/Si–OH minerals	((6 + 8)/7)			
	(Pour et al.^32^; van der Meer et al.^75^)			
Epidote/chlorite/amphibole	(6 + 9)/(7 + 8)			
	(van der Meer et al.^75^)			
Amphibole/MgOH	(6 + 9)/8 and 6/8			
	(van der Meer et al.^75^)			
Carbonate	13/14			
	(van der Meer et al.^75^)			
Sericite/Muscovite/Illite/Smectite Al/Fe–OH	(5 + 7)/6			
	(Pour et al.^32^; van der Meer et al.^75^)			
Alunite/kaolinite/pyrophyllite	(4 + 6)/5			
	(van der Meer et al.^75^)			

Due to its specified SWIR coverage (ASTER bands 5:7 are only covered by band 7 for L8, band 12 for Sentinel 2, and band 9 for ALI), ASTER data could specifically highlight areas invaded by other hydroxyl-bearing alterations (Table 2) compared to the general extraction of Sentinel 2 (11/12), L8 (6/7), and ALI (8/9). Consequently, relative absorption band depth (RBD) is applied to ASTER data for highlighting rocks enriched with alunite/kaolinite/pyrophyllite ((4 + 6)/5), sericite/muscovite/illite/smectite ((5 + 7)/6), chlorite/epidote ((7 + 9)/8), and Si–OH ((6 + 8)/7) minerals^32^.

#### Directed principal component analysis (DPCA)

DPCA, (also known as feature-oriented principal component analysis^61^ or selective principal component analysis^16,66,67^) is a robust feature extraction method that depends mainly on analyzing the principal component eigenvector loadings of feature interrelated bands^12,32,68^. The main difference between PCA and DPCA is that the latter utilized mostly a group (subset) of bands to highlight a certain feature, i.e. the selected bands to be analyzed are more or less related to the target. According to the spectral range of each band and the desired alteration type, bands are selected and analyzed using DPCA. Strong eigenvector loadings of different signs (positive, bright pixels, or negative, dark pixels) show the contribution of that component in highlighting the type of alteration. According to crosta technique applied to Landsat TM (using bands 1, 4, 5, and 7) for highlighting hydroxyl-bearing minerals^69^ (diagnostic spectral characteristics in the 2.10–2.28 μm range), the four datasets could disclose rocks enriched in these minerals (OH-bearing) utilizing the same spectral ranges. Consequently, Sentinel 2 bands 2, 8a, 11, 12, and Landsat OLI bands 2, 5, 6, and 7, and ALI bands 2, 6, 8, and 9 are utilized for revealing rocks enriched with OH-bearing minerals using DPCA. For ASTER data, band 1 (instead of the 0.48 nm range) is used together with bands 3, 4, and 7 for this analysis. Investigation of iron oxides (hematite, goethite, and limonite) through the USGS spectral library^70^ revealed diagnostic absorption features for ferric iron closer to 0.45–0.90 µm and 0.90–1.2 µm for ferrous iron (approximately 0.4–1.1 µm for both). Thus, iron oxides could be identified by incorporating VNIR bands as reported by Loughlin^68^ for Landsat TM bands 1, 3, 4, and 5. Consequently, iron oxide bearing rocks are analyzed through ASTER bands 1, 2, 3, and 4, Sentinel 2 bands 2, 4, 8a, 11, Landsat OLI bands 2, 4, 5, and 6, and ALI bands 2, 4, 6, and 8. This study applies the DPCA technique to the data obtained from BRs aiming to enhance the hydrothermal alteration targets^61^. Furthermore, we compared the reliability of the extracted alteration zones from DPCA transformation with the radiometric data findings and field observations. For ASTER data, four key band ratios are implemented for DPCA to highlight rocks enriched with ferric/ferrous iron oxides (2/1, 4/2), Al/Fe–OH ((5 + 7)/6), and Mg–Fe–OH ((7 + 9)/8) minerals. Landsat 8 band ratios (4/2, 6/4, 6/5, and 6/7) are used as inputs for DPCA to manifest rocks enriched in ferric /ferrous and Fe-OH iron oxides, ferrous oxides, ferric oxides, and hydroxyl-bearing minerals (Al–OH and Fe, Mg–OH). Similarly, DPCA was applied to Sentinel 2 (4/2, 11/4, 11/8a, and 11/12) and ALI (4/2, 8/4, 8/6, and 8/9) band ratios.

#### Constrained Energy Minimization method (CEM)

CEM is a spectral mapping technique for the localization of minerals based mainly on employing a finite impulse response filter to allow only the desired targets to pass through while minimizing the output energy of any background noise according to a particular constraint^12^. CEM is a well-known approach and widely utilized with ASTER data^12,71–73^ in geological and hydrothermal alteration mappings. To confirm the existence of alteration minerals and for more specifications, the CEM algorithm was applied to the potential ASTER SWIR bands^12,71–73^ to verify and specify the dominant hydroxyl-bearing minerals (within the detected hydroxyl-altered zones). Utilizing laboratory reflectance spectra of muscovite, illite, kaolinite, pyrophllite, montmorillonite, epidote, and chlorite derived from the USGS spectral library, pixels enriched with these minerals are highlighted and visually compared with the previous results and the geological context of the study area. These minerals are selected as they are among the common hydrothermal alteration index minerals for propylitic alteration (e.g. chlorite, epidote), argillic alteration (e.g. kaolinite, illite, Montmorillonite), and phyllic alterations (e.g. Pyrophyllite). Additionally, all of them are OH-bearing minerals and could be used to check and verify the OH-bearing alteration zones in the current research.

### Airborne geophysical data and processing techniques

**Figure 4:**
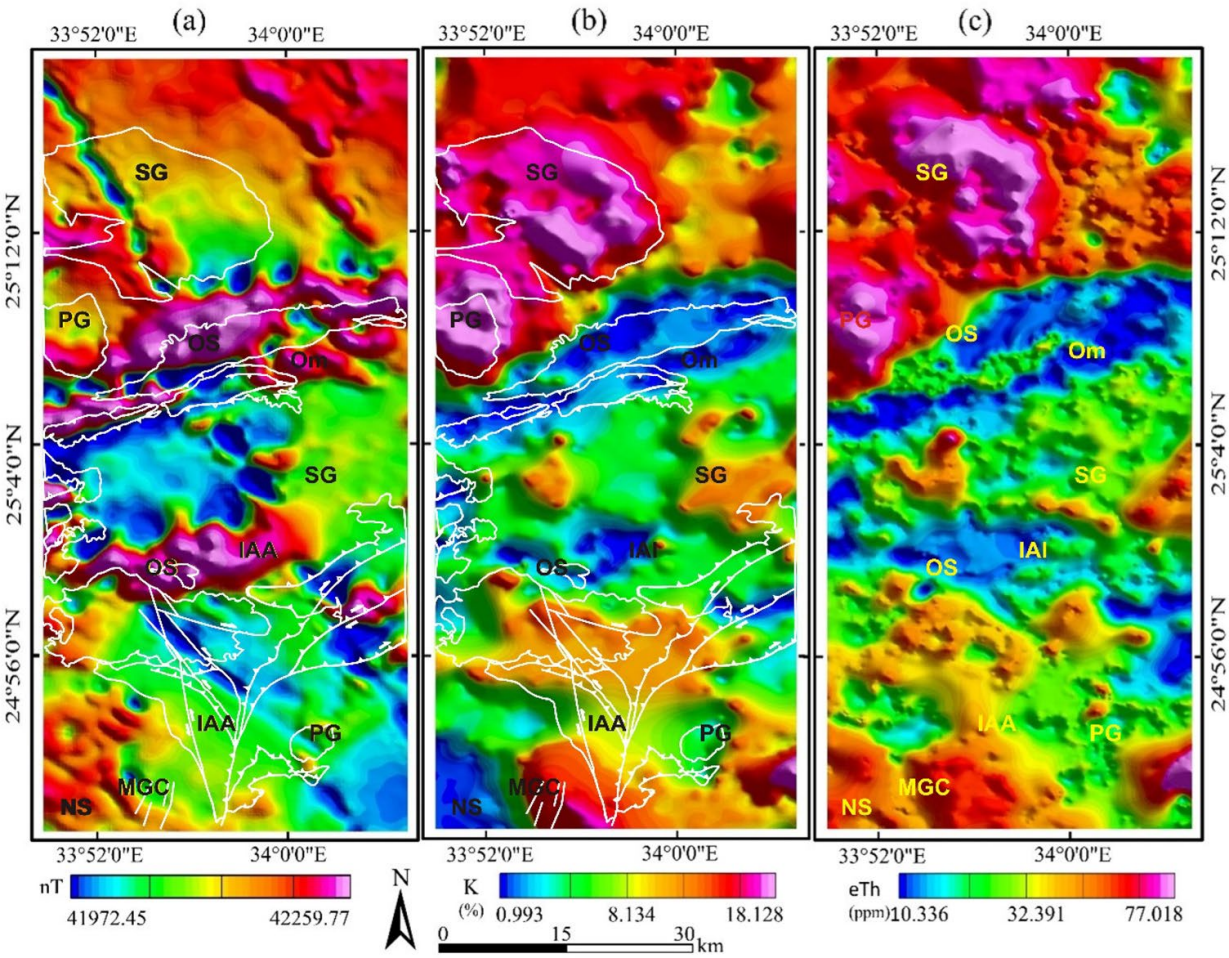
Airborne geophysical data (**a**) Reduction to pole (RTP) map, (**b**) potassium concentration (K) map, (**c**) Equivalent thorium concentration (eTh). (Created by Geosoft Oasis Montaj 2015 v. 8.3.3 software; https://www.seequent.com/help-support/oasis-montaj/).

The data used in this study is merged with the airborne magnetic and gamma-ray spectrometric survey performed by the Aero Service Division of Western Geophysical Company of America in 1983. The magnetic and radiometric data are available in the form of reduction to pole (RTP), potassium counts (K), and equivalent thorium (eTh) concentrations (Fig. 4a–c, respectively). The data maps provided a clearer depiction of the boundaries between rock units and a more direct delineation of the magnetic and radiometric lineaments in the area under investigation, enabling for even more accurate identification of the types and positions of various structures (e.g., faults, contacts, and shear zones).

Since the main purposes of using the airborne geophysical data are to confirm the detected alteration pattern and map its structural framework, the following approaches are used to achieve this target: (a) Center for Exploration Targeting (CET) porphyry analysis, which is a new filter that contains a three stage sequential procedures (circular feature detection, boundary enhancement and tracing) to detect the porphyry magnetic signature of the hydrothermally altered rocks^37^; (b) eTh/K ratio, which is derived from potassium and thorium concentration maps (Fig. 4b,c) to detect zones of potassic, phyllic and prophylitic hydrothermal alteration^40^; and (c) 3D Euler deconvolution^74^, involves the orthogonal gradients of the potential field in the horizontal (x,y) and vertical (z) directions and can be expressed as $\left(x-{x}_{0}\right)\frac{\partial t}{\partial x}+\left(y-{y}_{0}\right)\frac{\partial t}{\partial y}+\left(z-{z}_{0}\right)\frac{\partial t}{\partial z}=\left(B-T\right)N$, where N is the structural index, B is the field base level, T is the magnetic field at x, y and z coordinates. The method applied on the RTP magnetic data (Fig. 4a) to map the structural framework of the altered areas and estimate their depths.

## Results

### Geological mapping

FCCs built from ASTER (9/4/1 in RGB) and Sentinel 2 (12/11/2 in RGB) data separate serpentinite rocks in a dark black color compared to greenish-yellow metavolcanics (shown in dark red color in the case of L8 (7/5/3 in RGB) and ALI (9/6/3 in RGB), as shown in Fig. 3. The selected bands for the FCCs are more or less around the same spectral range. For example, in the red channel of the given FCCs (displayed in Fig. 3), ASTER band 9, Sentinel 2 band 12, Landsat 8 band 7, and ALI band 9 are all representing SWIR of approximately 2.2 µm (Table 1) that is very useful in geological discriminations. Similarly and after several trials, and checking previous studies related to the lithological mapping with these sensors^1,10^, the RGB composites for each sensor were selected. For lithological mapping, PCA is applied to all the bands of each sensor (shown in Table 1) to discriminate the lithological targets and compare them with previous geological maps and our field observations. ASTER PCs (2/1/3 in RGB) show serpentinites in a strong, golden yellow colour (confirmed in a pinkish-white color in PCs 2/1/3 in RGB of L8) that could be easily delineated. Metagabbroic rocks are shown in a greenish colour for ASTER and L8 PCs. Unambiguous discrimination of the weathered syn-tectonic granites (greenish) from wadi deposits (greyish) is presented through Sentinel 2 PCs (2/1/3 in RGB) and confirmed through ALI PCs (3/2/1 in RGB) results (sky blue color for syn-tectonic granites). The sharp distinction between metavolcanics and serpentinites is also displayed through ALI and Sentinel 2 results. Separation of volcaniclastic metasediments (representing the mélange matrix) from the other rock units may be difficult as they contain spectral signatures of the neighboring rock units. Overall and after careful visual interpretation with previous geological maps and intensive field work, our results revealed that the four datasets are eligible (almost similar effectiveness) for highlighting all the exposed rock units (Fig. 3).

### Hydrothermal alteration mapping

**Figure 5:**
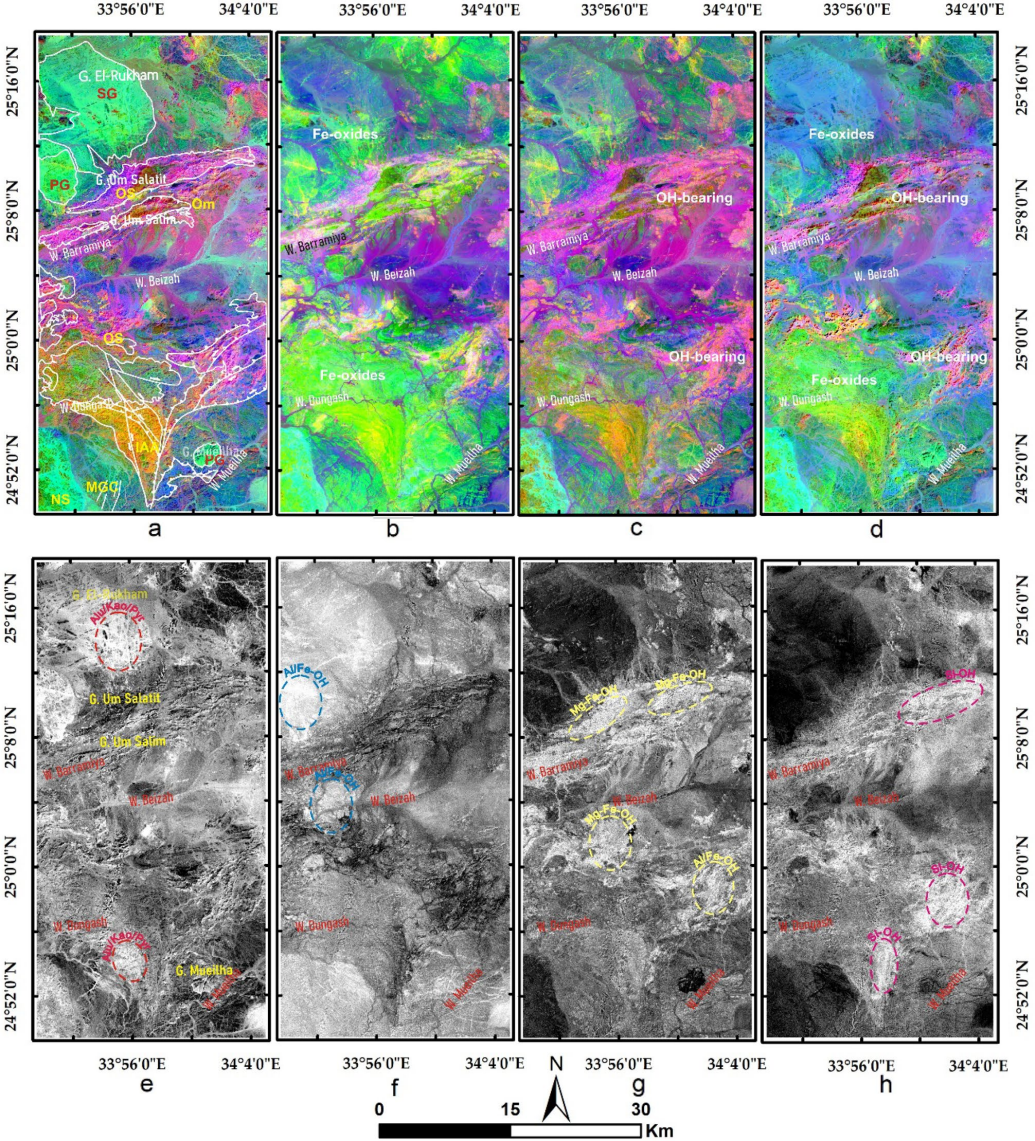
Depiction of the general hydrothermal alterations using combinations of (**a**) ASTER, (**b**) Landsat 8, (**c**) Sentinel 2 and (**d**) ALI. Relative absorption band depth (RBD) results for highlighting (**e**) Alunite/Kaolinite/Pyrophyllite, (**f**) Al/Fe–OH, (**g**) Mg–Fe–OH and (**h**) Si–OH bearing rocks as bright pixels.

Results of the main hydrothermal alteration pattern (Table 3) revealed that the four-color composites of ASTER (4/7, 2/1, 4/3), Sentinel 2 (11/12, 4/2, 1/8), Landsat OLI (6/7, 4/2, 6/5),and ALI (8/9, 4/1, 8/6) are visually comparable, especially the hydroxyl-bearing alteration (Red channel), which exhibits almost the same pattern as shown in Fig. 5a–d. Compared to the geological maps, hydroxyl-bearing alterations are mainly confined to serpentinites. Metavolcanics are affected by iron oxides and hydroxyl-bearing minerals. Generally, iron oxides are mainly represented by blue and green colors and are restricted mainly to ophiolitic mélange represented by volcaniclastic metasediments, weathered syn-tectonic granites, and quaternary deposits. The known mineral deposits (Fig. 3a) are mostly located within the altered zones. This, in turn, confirms the eligibility of the four sensors for depicting the main hydrothermal alteration zones that are further verified and specified with other adopted techniques.

**Table 3:** Thresholds, and anomalies of the detected hydrothermal alterations (and their intersections), using band ratios of the four utilized datasets.

BR	Mean	Standard deviation	Threshold	Anomalies (km^2^)	Overestimated area
ASTER 4/7	116.73	62.20	178.93	252.1244	148.40755
ASTER 2/1	117.26	57.04	174.3	234.0096	183.1392
ASTER 4/3	132.89	57.37	190.26	217.644	150.767575
S2 11/12	128.86	58.06	186.92	240.2932	136.57635
S2 4/02	86.44	44.71	131.15	209.1736	158.30321
S2 11/8	137.46	56.01	193.47	211.134	144.257575
L8 6/7	111.77	55.52	167.29	219.0264	115.30955
L8 4/2	176.06	60.80	236.86	350.7412	299.8708
L8 6/5	136.79	60.80	197.59	216.6624	149.785975
ALI 8/9	118.64	51.40	170.04	205.8885	102.17165
ALI 4/1	172.55	43.08	215.63	234.5877	183.717313
ALI 8/6	169.39	53.55	222.94	240.5781	173.701675

Intersections	OH	iron oxides	Ferrous oxides		
ASTER	252.1244	234.0096	217.644		
Sentinel 2	240.2932	136.57635	211.134		
L8	219.0264	115.30955	216.6624		
ALI	205.8885	102.17165	240.5781		
ASTER + Sentinel 2	146.57486	101.63106	114.72246		
ASTER + L8	135.4027	87.13305	113.3808		
ASTER + ALI	144.8665	73.12402	118.516675		
four	103.71685	50.870387	66.876425

It should be emphasized that relative band depths could highlight a wide range (category or group of minerals) of hydrothermal alteration and cannot specify a certain mineral when compared with spectral localization methods (e.g. CEM). For example, RBD could highlight the existence of phyllosilicates clay minerals (e.g. alunite/kaolinite/pyrophyllite)^75^ but cannot specify a certain mineral like CEM that could highlight a single mineral based on its spectral signature (reflectance or absorption spectra). Results of the RBD method applied to ASTER show the distribution of phyllosilicates clay minerals (alunite/kaolinite/pyrophyllite), Al/Fe–OH, Mg–Fe–OH, and Si–OH minerals as shown in Fig. 5e–h. A reasonable allocation of these minerals is notable concerning the exposed lithologies. For instance, alunite/kaolinite/pyrophyllite is mainly concentrated on intensively weathered granitic rocks in the northern part of the study area. Similarly, Al/Fe–OH-enriched minerals (mostly sericite/muscovite/illite/smectite) are associated with syntectonic granite and ferruginous Nubian sandstone. Alternatively, chlorite/epidote minerals are approximately congruous with serpentinite rocks. Si–OH minerals are extensively represented over the ophiolitic mélange, metavolcanic, and serpentinites.

**Figure 6:**
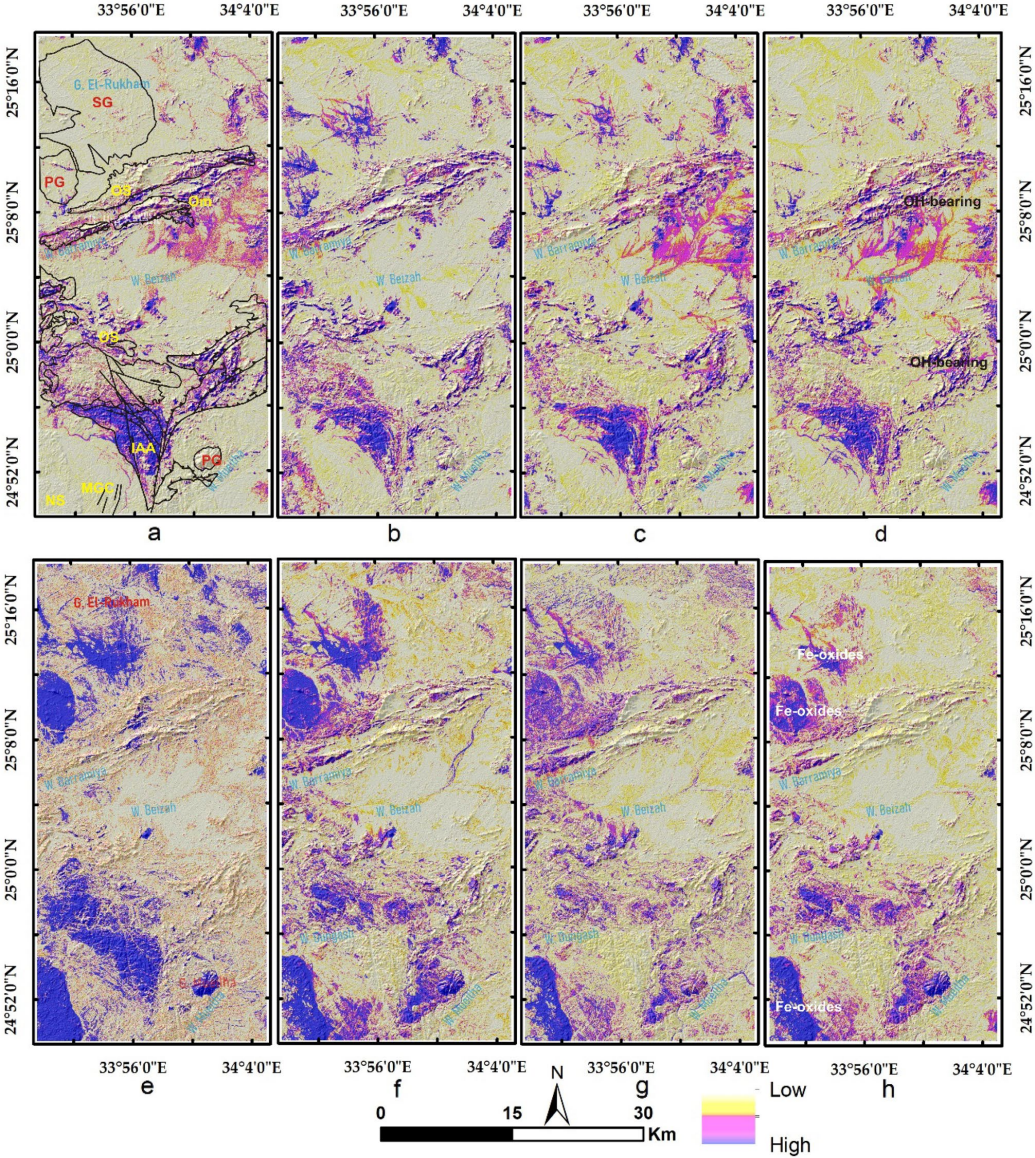
Pseudocolor ramps representing rule images of (**a**) ASTER DPC4, (**b**) Landsat 8 DPC3, (**c**) Sentinel 2 DPC4 and (**d**) ALI DPC3 showing rocks enriched with hydroxyl-bearing minerals. Pseudocolor ramps representing rule images of (**e**) ASTER DPC2, (**f**) Landsat 8 DPC3, (**g**) Sentinel 2 DPC3 and (**h**) ALI DPC3 showing iron oxides' bearing rocks.

In Table 4, different kinds of alterations are shown based on how much the input band or band ratio contributed to the eigenvector matrix of DPCA. Scrutinizing the magnitude and sign of eigenvector loadings extracted from DPCA was extremely useful for delineating alteration minerals groups in the four datasets. For instance, in ASTER DPCA of bands 1, 3, 4, and 7, the first DPC does not reveal any specific information for any type of hydrothermal alteration, as all the eigenvector loadings for the four input bands are negative (Table 4). On contrary, DPC 4 has strong positive loadings for B7 (0.96) and very small positive or negative loading for bands 1, 3, and 4. Thus, DPC4 (Fig. 6a) strongly reveals the desired target from this combination (hydroxyl-bearing minerals). For Landsat 8, DPC 1 is less informative due to the dominance of positive loadings for all the input bands. DPC 3 (Fig. 6b) is better than DPC 2 and DPC 4 in distinguishing hydroxyl-bearing minerals due to its strong loadings for band 7 (0.67) compared to the other components (Table 4). In the Sentinel 2 findings, DPC1 doesn't introduce any unique information due to the dominance of negative loadings for all the input bands. DPC 2 has moderate loadings of approximately all alteration mineral groups and thus could not strongly highlight any of them. DPC 3 could reasonably highlight iron oxides due to the negative loadings in band 2 and the positive ones in band 8a; however, discriminating hydroxyl-bearing minerals is extremely difficult due to the weak loadings in bands 11 and 12. DPC 4 (Fig. 6c) could distinguish hydroxyl-bearing minerals due to strong loadings in bands 11 and 12. Similarly, ALI DPC 3 (Fig. 6d) is the best to highlight OH-enriched minerals due to the high loadings in band 9 compared to the same band loadings in the other components. Consequently, our results are greatly harmonized with the previous studies^7,76–78^ that accentuated the presence of the altered anomalies in the noisiest components (the third or the fourth), and the current research revealed that the best DPCs for disclosing hydroxyl-bearing minerals are ASTER DPC 4, Sentinel 2 DPC 4, L8 DPC 3, and ALI DPC 3. In the same way, by analyzing the eigenvector matrix to distinguish iron oxides' bearing rocks, ASTER DPC 2 (Fig. 6e), L8 DPC 3 (Fig. 6f), Sentinel 2 DPC 3 (Fig. 6g), and ALI DPC 3 (Fig. 6h) are the most informative components. For Sentinel 2, L8, and ALI, the desired wavelength (b2, approximately 0.48 µm) for distinguishing iron oxides has a strong loading in DPC 3; despite the lack of this wavelength in ASTER, DPC 2 was the best component, exhibiting the strongest loading (0.71) in band 1.

**Table 4:** The Eigenvector matrix values of bands, band ratio indices, and Relative Band Depths utilized for DPCA using the four datasets to highlight various alteration types.

Hydroxyl-bearing minerals				
Eigenvector (ASTER)	B1	B3	B4	B7
DPCA 1	− 0.75661	− 0.63646	− 0.14441	− 0.04012
DPCA 2	− 0.6277	0.642843	0.419598	0.129167
DPCA 3	− 0.18274	0.425821	− 0.86022	− 0.21286
DPCA 4	0.01222	− 0.01853	− 0.25122	0.967676

Eigenvector (S2)	B2	B8a	B11	B12
DPCA 1	− 0.2338	− 0.49105	− 0.61177	− 0.57441
DPCA 2	− 0.69378	− 0.51409	0.290978	0.411961
DPCA 3	− 0.68009	0.68397	− 0.04489	− 0.26009
DPCA 4	− 0.03857	0.163603	− 0.73421	0.65779

Eigenvector (L8)	B2	B5	B6	B7
DPCA 1	0.18568	0.510246	0.630871	0.554232
DPCA 2	0.501779	0.677057	− 0.30644	− 0.44261
DPCA 3	0.390401	− 0.12282	− 0.61093	0.677691
DPCA 4	0.749219	− 0.51591	0.367229	− 0.19405

Eigenvector (ALI)	B2	B6	B8	B9
DPCA 1	− 0.20929	− 0.43703	− 0.64853	− 0.58704
DPCA 2	− 0.63163	− 0.61918	0.290954	0.364706
DPCA 3	0.018854	0.071684	− 0.69859	0.711673
DPCA 4	− 0.74625	0.648453	− 0.08204	− 0.12607

Iron oxides				
Eigenvector (ASTER)	B1	B2	B3	B4
DPCA 1	0.567561	0.660388	0.479535	0.108667
DPCA 2	0.715437	− 0.138838	− 0.569369	− 0.380385
DPCA 3	0.293986	− 0.282978	− 0.161860	0.898497
DPCA 4	− 0.282127	0.681568	− 0.647817	0.190268

Eigenvector (S2)	B2	B4	B8a	B11
DPCA 1	0.265453	0.498315	0.529967	0.632734
DPCA 2	0.493719	0.511591	0.102312	− 0.69574
DPCA 3	0.586513	0.04036	− 0.73503	0.337798
DPCA 4	0.584618	− 0.6988	0.410368	− 0.03864

Eigenvector (L8)	B2	B4	B5	B6
DPCA 1	0.206659	0.455838	0.554507	0.664850
DPCA 2	0.388539	0.477569	0.323917	− 0.718362
DPCA 3	0.778045	0.071257	− 0.590603	0.201883
DPCA 4	− 0.448303	0.747705	− 0.488660	0.034262

Eigenvector (ALI)	B2	B4	B6	B8
DPCA 1	0.238313	0.483415	0.482739	0.690275
DPCA 2	0.412695	0.523427	0.267211	− 0.69592
DPCA 3	0.768503	− 0.04186	− 0.60948	0.190231
DPCA 4	− 0.42696	0.700415	− 0.5693	0.055022

Ferric/ferrous iron oxides, Al/Fe–OH, and Mg–Fe–OH bearing minerals				
Eigenvector (ASTER)	B2/B1(ferrous)	B4/B2 (ferric)	((B7 + B9)/B8) Mg–Fe–OH	((B5 + B7)/B6) Al/Fe–OH
DPCA 1	0.000343	0.000055	− 0.70566	0.708556
DPCA 2	− 0.00023	0.000046	0.708556	0.705655
DPCA 3	0.999274	0.038094	0.000405	− 0.000084
DPCA 4	0.038094	− 0.99927	0.000009	0.000069

Eigenvector (S2)	B4/B2 (iron oxides)	B11/B4 (ferrous)	B11/B8a (ferric)	B11/B12 (OH)
DPCA 1	0.283757	0.8326	0.474493	− 0.0334
DPCA 2	0.946776	− 0.17216	− 0.26754	− 0.04896
DPCA 3	0.076989	− 0.06169	0.131627	0.986378
DPCA 4	0.131033	− 0.52281	0.828223	− 0.15344

Eigenvector (L8)	B4/B2 (iron oxides)	B6/B4 (ferrous)	B6/B5 (ferric)	B6/B7 (OH)
DPCA 1	− 9.999	− 0.00275	− 0.00097	− 0.000032
DPCA 2	0.00286	− 0.86436	− 0.50152	0.036844
DPCA 3	− 0.00012	0.014032	0.049184	0.998691
DPCA 4	0.000543	− 0.50268	0.863746	− 0.03548

Eigenvector (ALI)	B4/B2 (iron oxides)	B8/B4 (ferrous)	B8/B6 (ferric)	B8/B9 (OH)
DPCA 1	− 0.00176	0.072038	0.9974	0.000685
DPCA 2	0.001892	− 0.9974	0.072042	− 0.00029
DPCA 3	− 0.00933	− 0.00035	− 0.00068	0.999956
DPCA 4	0.999953	0.002011	0.001617	0.009327

**Figure 7:**
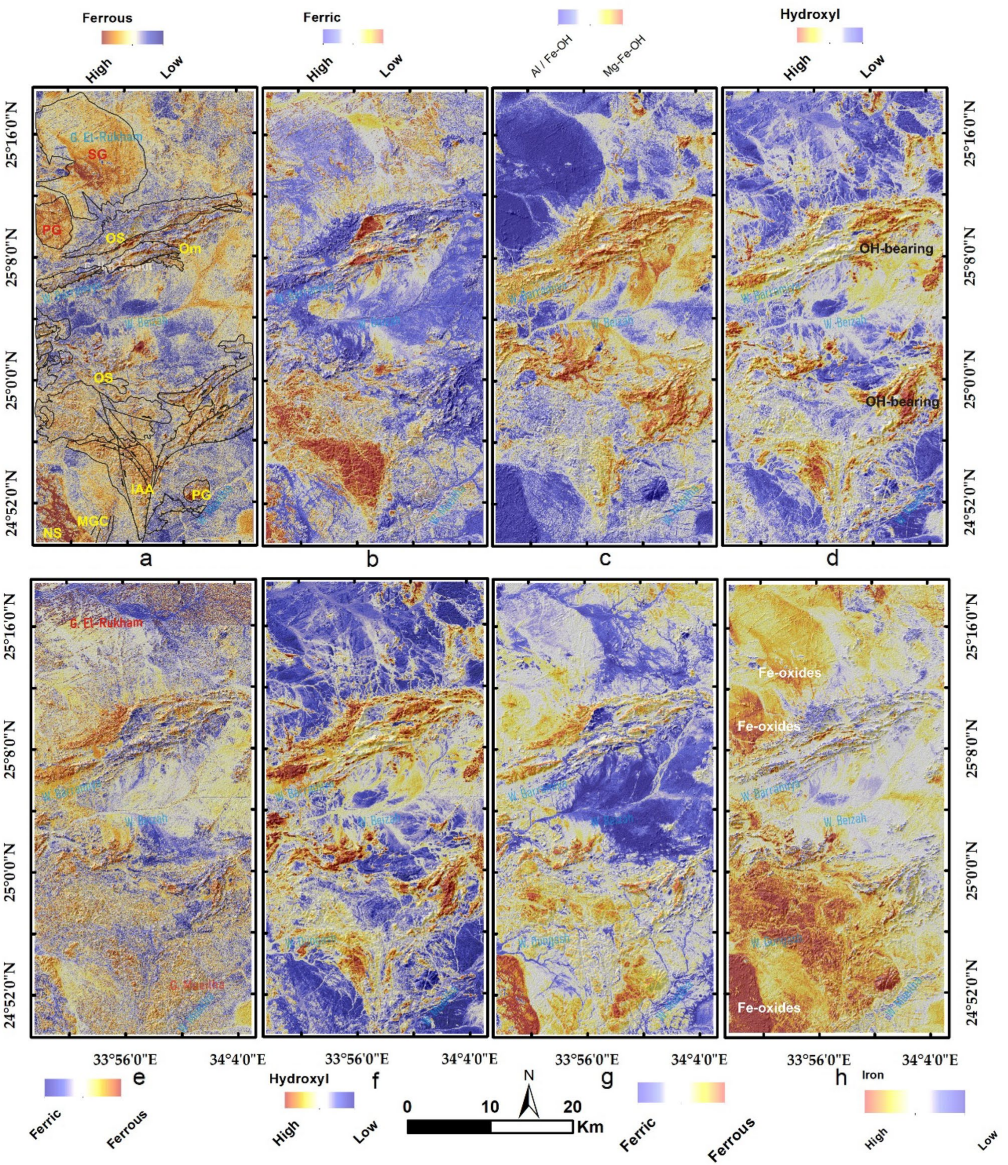
Pseudocolor ramps representing rule images of (**a**) ASTER DPC3, (**b**) ASTER DPC4, (**c**) ASTER DPC1, (**d**) Sentinel 2 DPC3 (**e**) Sentinel 2 DPC4, (**f**) Landsat 8 DPC4, (**g**) Landsat 8 DPC4 and (**h**) ALI DPC4 showing lithological units affected by various types of hydrothermal alterations within the study area.

DPCA was also applied to informative band ratio indices to specify the alteration types^61^. For ASTER data, the inputs were 2/1, 4/2, ((5 + 7)/6), and ((7 + 9)/8) to highlight rocks enriched with ferric/ferrous oxides, Al/Fe-OH, and Mg-Fe-OH. In DPC 3, ferrous oxides’ bearing rocks (Fig. 7a) could be mapped due to the strong contribution of the B2/B1 ratio input (0.99) compared to the other inputs. DPC 4 discriminates lithological units enriched with ferric iron (Fig. 7b) due to the strong negative loadings in B4/B2. Our results revealed that DPC 1 (Fig. 7c) is extremely useful in discriminating Al/Fe–OH rich minerals (5 + 7)/6) from Mg–Fe–OH enriched minerals ((7 + 9)/8). In this way, ASTER can not only detect hydroxyl-bearing minerals but also classify them as Al/Fe–OH or Mg–Fe–OH minerals, reflecting the absolute superiority of ASTER data due to its SWIR bands. Moreover, using the CEM technique, some of these minerals could be nominated and specified. Results of Sentinel 2 DPCA revealed the sublimity of DPC 3 (Fig. 7d) in recognizing rocks enriched with OH-bearing minerals due to the strong contribution of the 11/12 band ratio (0.98). The differentiation between ferrous (11/4) and ferric (11/8a) rocks was clear in DPC 4 (Fig. 7e), where the first has reasonable negative loadings (− 0.52) compared to the strong positive loadings (0.82) of the second. Analyzing the DPCA results of L8 data revealed the superiority of DPC 3 (Fig. 7f) in the distinction of rocks enriched with OH-bearing minerals due to the strong contribution of the 6/7 band ratio (0.99). The distinction between ferrous (6/4) and ferric (6/5) was given through DPC 4 loadings (Fig. 7g). Similarly, ALI discriminates rocks enriched with OH-bearing minerals in DPC 3 and collectively highlights iron oxides in DPC 4 (Fig. 7h).

**Figure 8:**
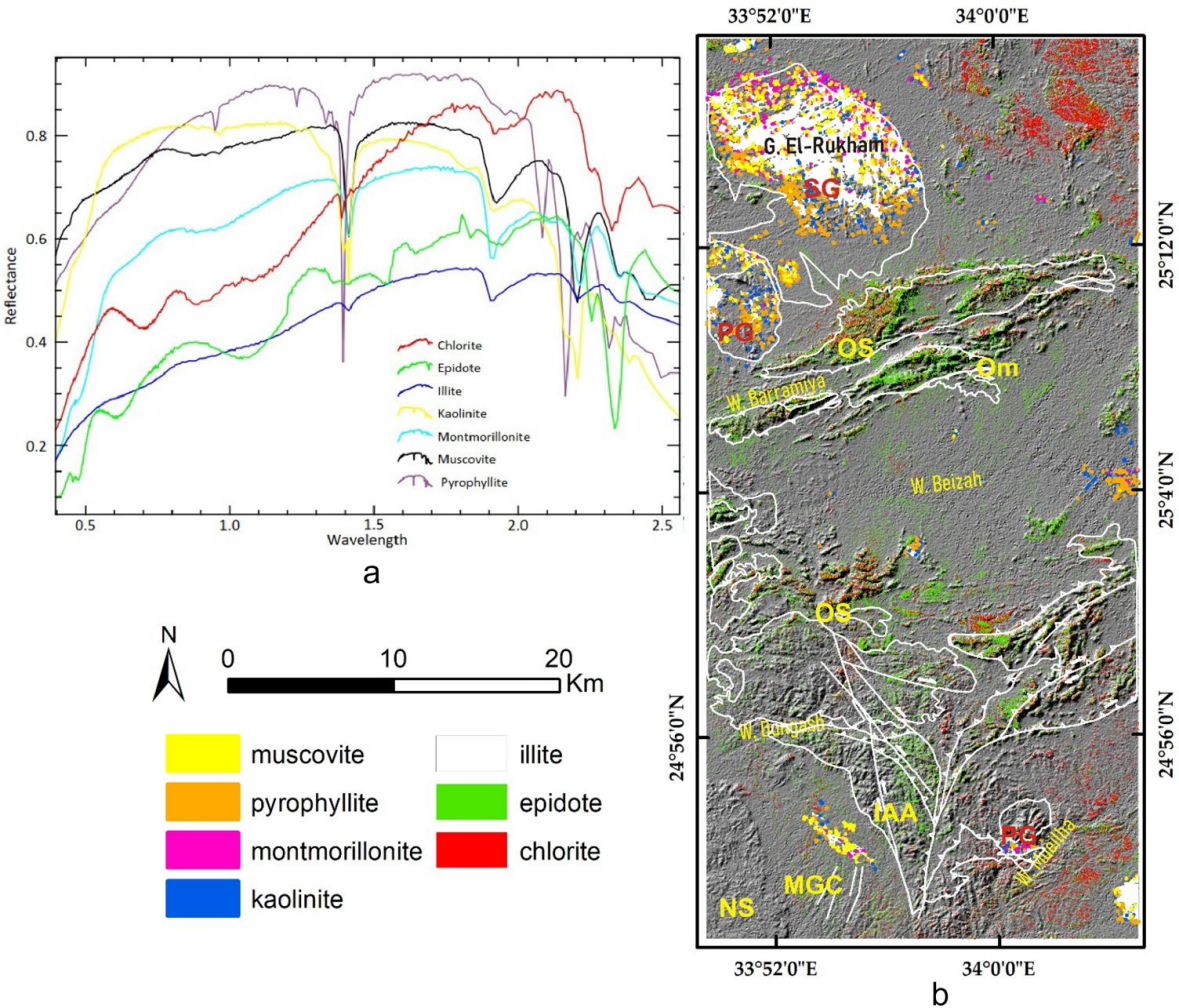
(**a**) Spectral curves (from USGS spectral library) of the minerals utilized in CEM analysis and (**b**) their distribution within the study area. (Created by ENVI v. 5.6.2. software; https://www.l3harrisgeospatial.com/Software-Technology/ENVI).

As a continuation of revealing the hydrothermal alteration patterns in the study area, the CEM technique is implemented in the current study to approximately specify alteration minerals in SWIR (using ASTER data) ranges. CEM results and as an additional validation method (Fig. 8) showed that approximately all the highlighted pixels for muscovite, illite, kaolinite, pyrophyllite, montmorillonite, epidote, and chlorite minerals are confined to the highlighted hydroxyl bearing zones (previously detected using DPCA and BRs). Moreover, chlorite and epidote are concentrated along serpentinite rocks. Clay minerals (e.g., montmorillonite, illite, and kaolinite) and muscovite are mostly restricted to weathered granitic rocks or wadi deposits. These results not only confirm the highlighted zones and the efficiency of the utilized techniques but also highlight ASTER data for specific alteration mapping. Therefore, results from the other three sensors were compared to ASTER using anomaly overlay analysis to select the best sensor to be combined with ASTER for further geological mapping. Integrating with ASTER data gives confirmation for OH-bearing altered zones (mostly associated with hydrothermal mineral deposits), where the other sensors have different bandwidths and wavelengths. However, before comparing the areal extents of all the detected hydrothermal alteration anomalies, a comprehensive airborne geophysical analysis was applied to evaluate the reality of these detected zones using airborne geophysical data, including the reduction to pole (RTP) map, potassium concentration (K) map, and equivalent thorium concentration (eTh) map.

**Figure 9:**
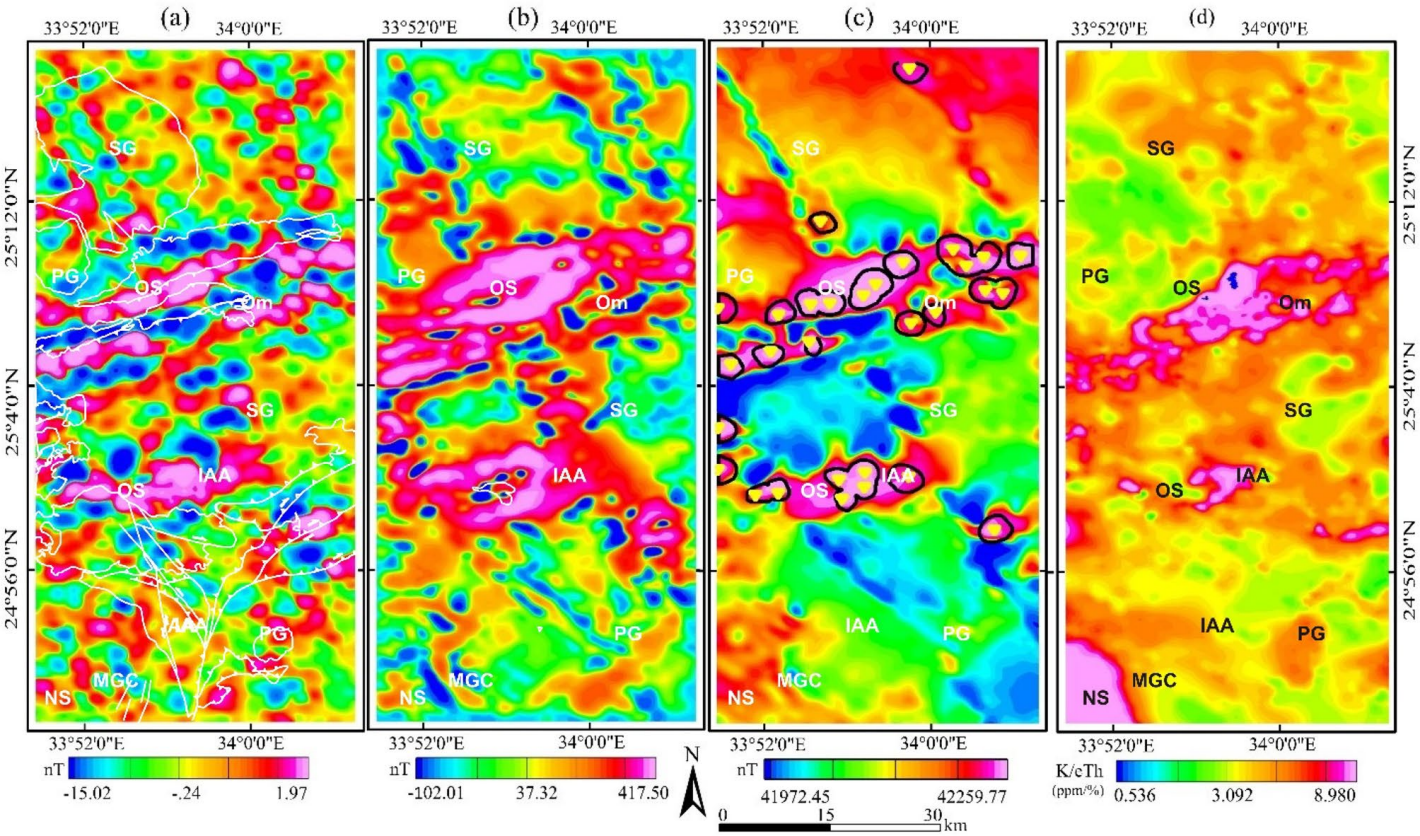
Hydrothermal alteration maps (**a**) Circular feature transform, (**b**) Amplitude contrast transform, (**c**) Boundary tracing of detected hydrothermal intrusions overlaid on RTP map, (**d**) K/eTh ratio map. (Created by Geosoft Oasis Montaj 2015 v. 8.3.3 software; https://www.seequent.com/help-support/oasis-montaj/).

By collaborating with magnetic and radiometric data, which are commonly used to map magnetic and radiometric features associated with hydrothermal alteration rocks. We could confirm the recording of shear zones and other related structures that more or less coincide with the formerly mentioned hydrothermal alterations in all interpreted maps of the investigated area, besides estimating their depths. Concerning aeromagnetic data, a set of algorithms for CET porphyry analysis are applied to the RTP magnetic map to locate the porphyry-like circular or semi-circular structures (intrusions) that are associated with hydrothermal alteration. The intrusions and/or the inner alteration zone are associated with high magnetic anomalies, while the outer ones are less magnetic. In the preliminary phase, the circular features and their centers in the study area are highlighted by applying the Circular Feature Transform (CFT) (Fig. 9a), and then the Amplitude Contrast Transform (ACT) is used to delineate the boundaries of the detected circular intrusions (Fig. 9b). The yields of the CET and ACT are utilized to outline the detected porphyry-like features (Fig. 9c). On the other hand, the aeroradiometric data in the form of equivalent potassium and thorium concentrations assist to discriminate between different rock types in the study area; for example, ophiolitic serpentine is characterized by low values in K and eTh maps while the highest values are related to syn-orogenic granitoids. In addition, the highest values of the K/eTh ratio map (Fig. 9d) characterized by pink color, reveal the areas of hydrothermal alterations. The visual inspections of Fig. 9a–d reveal the areas of much being hydrothermally altered, which are associated with ophiolitic serpentine and oriented in an ENE-WSW direction over Gabal Um Salatit and Gabal Um Salim (the Baramiya-Mubarak shear zone), which is well-explained in the following section.

### The structural context of the detected anomalies

**Figure 10:**
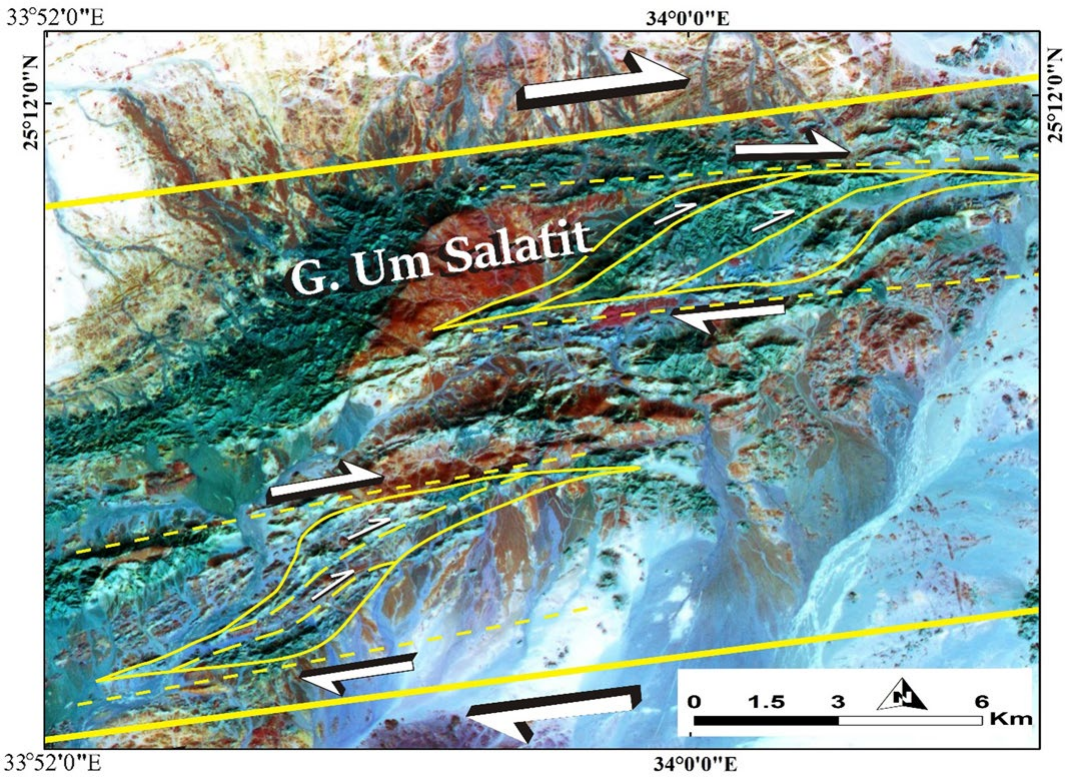
ASTER FCC 7/3/1 in RGB showing the dextral shear sense along Gebel Um Salatit.

Fowler^79^ hypothesized that structural discontinuities in the Central Eastern Desert, such as major transpressional and transtensional shear zones and moderately dipping thrusts, aided in the intrusion of syn-tectonic granitoid intrusions, many of which are linked with hydrothermal activity and mineralization^80–82^. Among these structures, the northwest transpressional Najd-style and its conjugate Barramiya-Mubarak shear zone of the spectacular northeast trend and their second-and third-order extensional shears, which are genetically linked with ore deposits, particularly gold mineralization^29,81,83–86^. Gold mineralization is claimed in a variety of distinct rock types, including ophiolitic serpentinite, arc metavolcanics, gabbroic, and granitoid rocks^87^. The Baramiya-Mubarak shear zone is detected locally in the research region at Gabal Um Salatit, which is composed of ophiolitic rocks and has a dextral displacement in the ENE–WSW direction (Fig. 10). Although this shear zone and its secondary fractures are not directly responsible for mineralization, they provide a pathway for the transformation of hydrothermal fluids, which are mainly responsible for the detected hydrothermal features in the current research.

**Figure 11:**
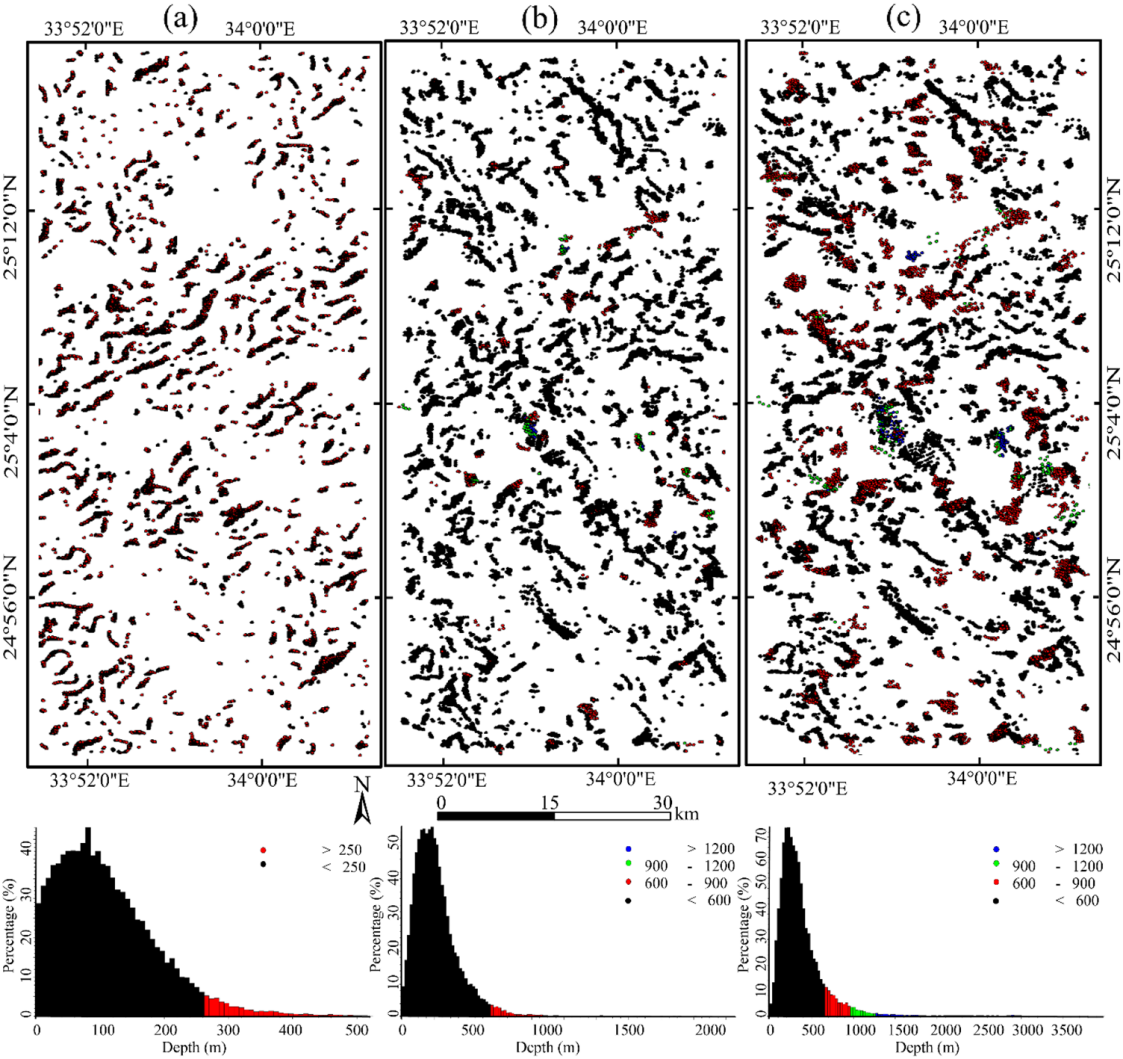
structural features and their depths by Euler deconvolution for, (**a**) Contact SI = 0.0, (**b**) dykes SI = 0.5, (**c**) faults SI = 1.0. (Created by Geosoft Oasis Montaj 2015 v. 8.3.3 software; https://www.seequent.com/help-support/oasis-montaj/).

To better assist in mapping structural features (key elements for hydrothermal alterations) of the investigated area and estimate their depths, the aeromagnetic RTP map (Fig. 4a) is used to perform the 3D Euler deconvolution method. The method was utilized with structural indexes 0, 0.5, and 1 for contacts, dykes, and faults, respectively. It is observed that the main tectonic trends in the area are oriented in NW–SE and ENE–WSW directions. The obtained depths are illustrated in Fig. 11a–c. A statistical analysis was performed to determine the prevailing depths. It is shown that most of the depths fall in the range of 0–600 m, indicating shallow sources, while the deeper depths up to 1500 m indicate moderate and deep depths of dykes and fault-like structures. In conclusion, the aeromagnetic data in the form of RTP combined with the Euler deconvolution clearly enhanced the appearance of the shear zone and other magnetic structures (contacts, dykes, and faults) that mostly constitute the pathways for hydrothermal fluids and gave an estimation for their depths.

## Field verification and petrographic investigations

**Figure 12:**
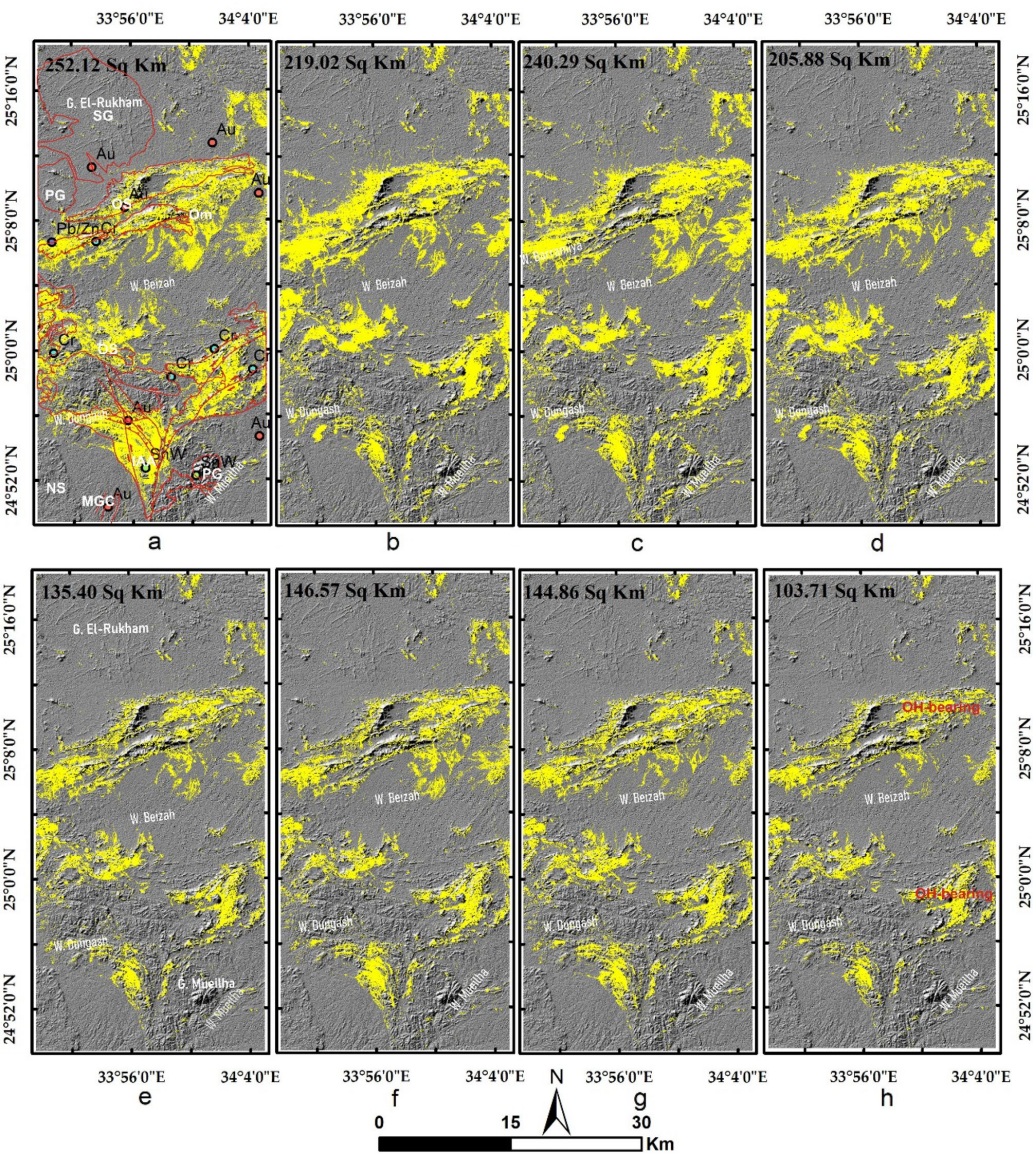
Areal differences of the rocks enriched with hydroxyl-bearing minerals (yellow color) detected utilizing (**a**) ASTER, (**b**) Landsat 8, (**c**) Sentinel 2, and (**d**) ALI. Intersected results using (**e**) ASTER and Landsat 8, (**f**) ASTER and Sentinel 2, (**g**) ASTER and ALI, and (**h**) the four sensors dropped over shaded relief map.

**Figure 13:**
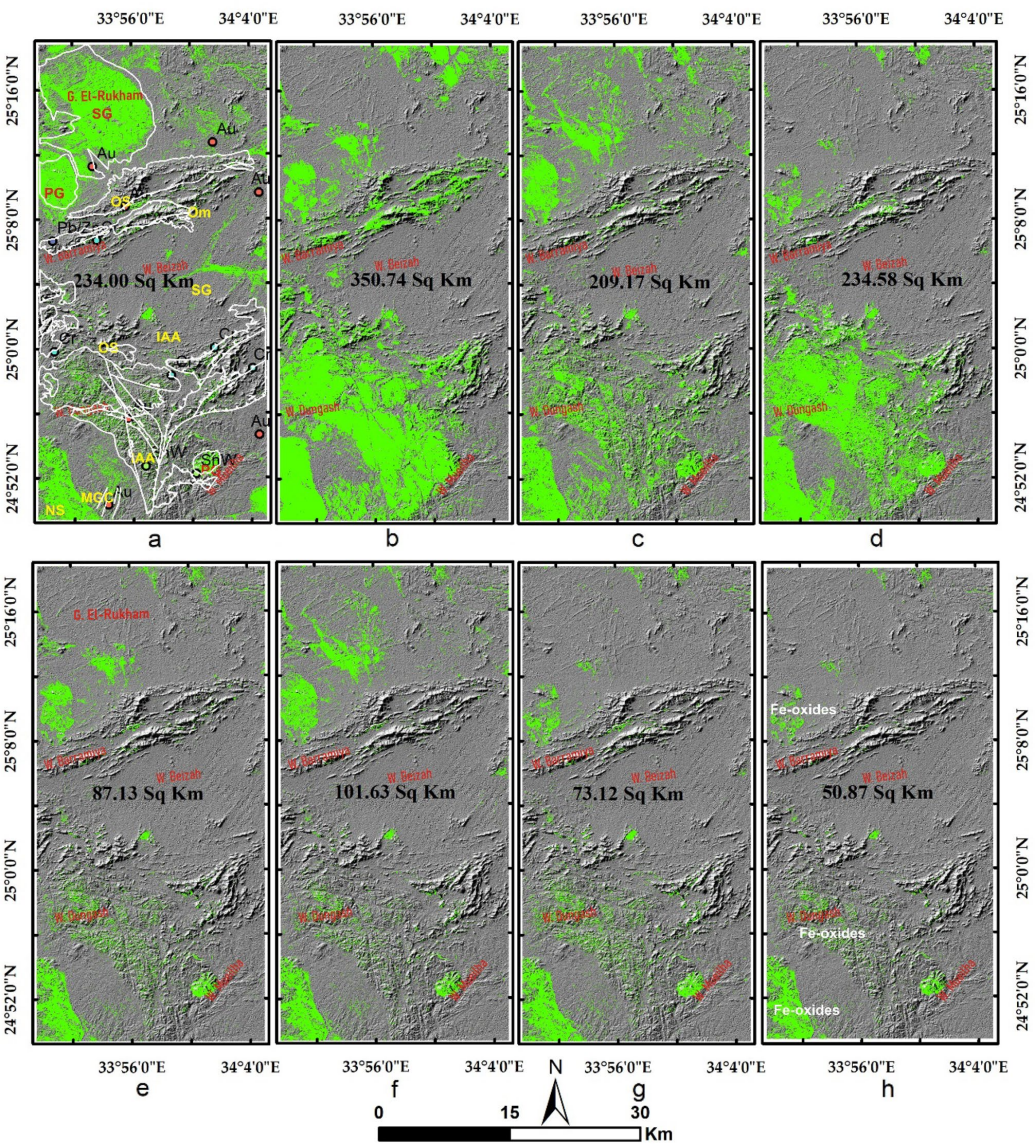
Areal differences of iron oxides’bearing rocks (green color) detected utilizing (**a**) ASTER, (**b**) Landsat 8, (**c**) Sentinel 2, and (**d**) ALI. Intersected results using (**e**) ASTER and Landsat 8, (**f**) ASTER and Sentinel 2, (**g**) ASTER and ALI, and (**h**) the four sensors dropped over shaded relief map.

**Figure 14:**
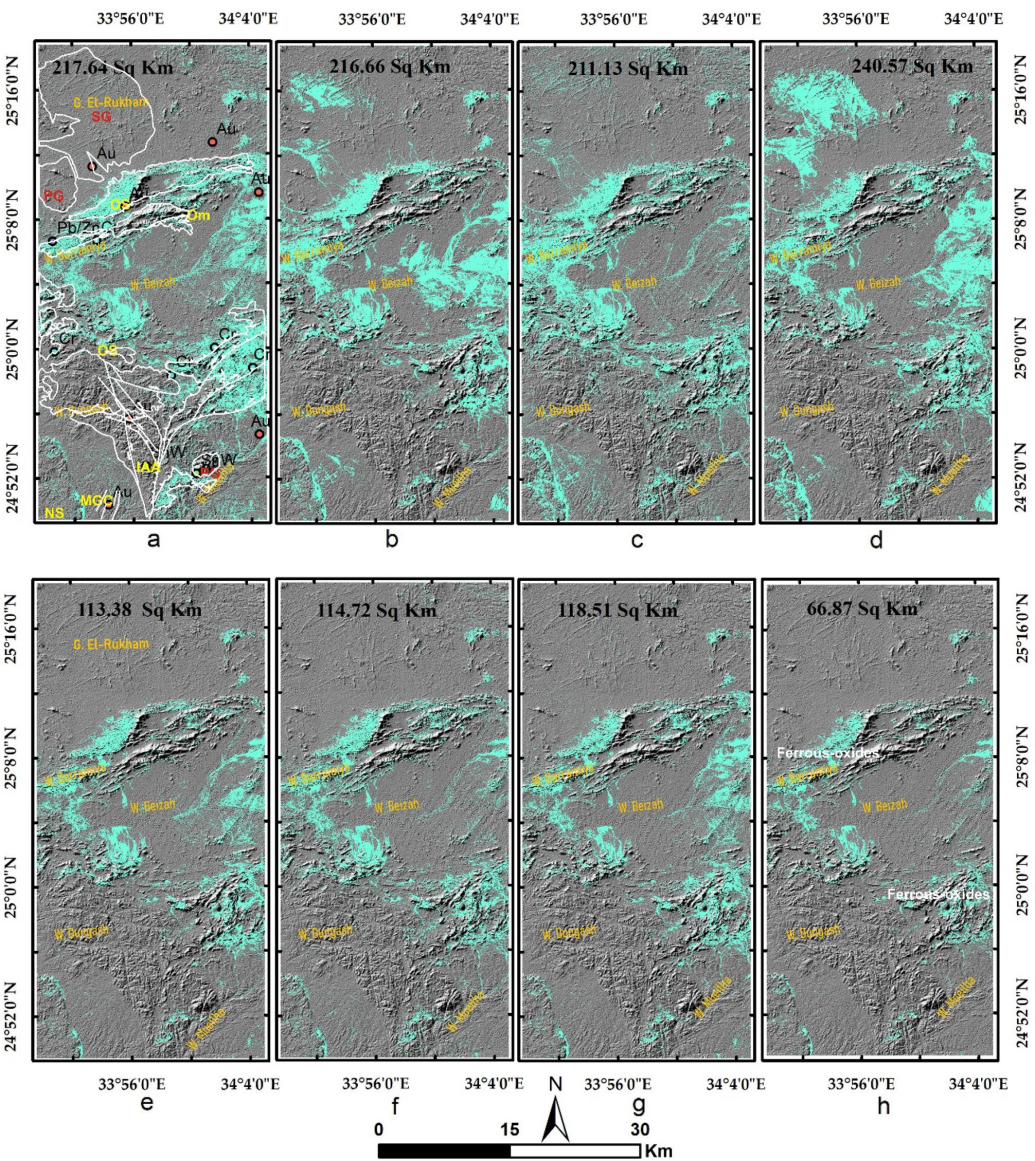
Areal differences of ferrous oxides’ bearing rocks (cyan color) detected utilizing (**a**) ASTER, (**b**) Landsat 8, (**c**) Sentinel 2, and (**d**) ALI. Intersected results using (**e**) ASTER and Landsat 8, (**f**) ASTER and Sentinel 2, (**g**) ASTER and ALI, and (**h**) the four sensors dropped over shaded relief map.

**Figure 15:**
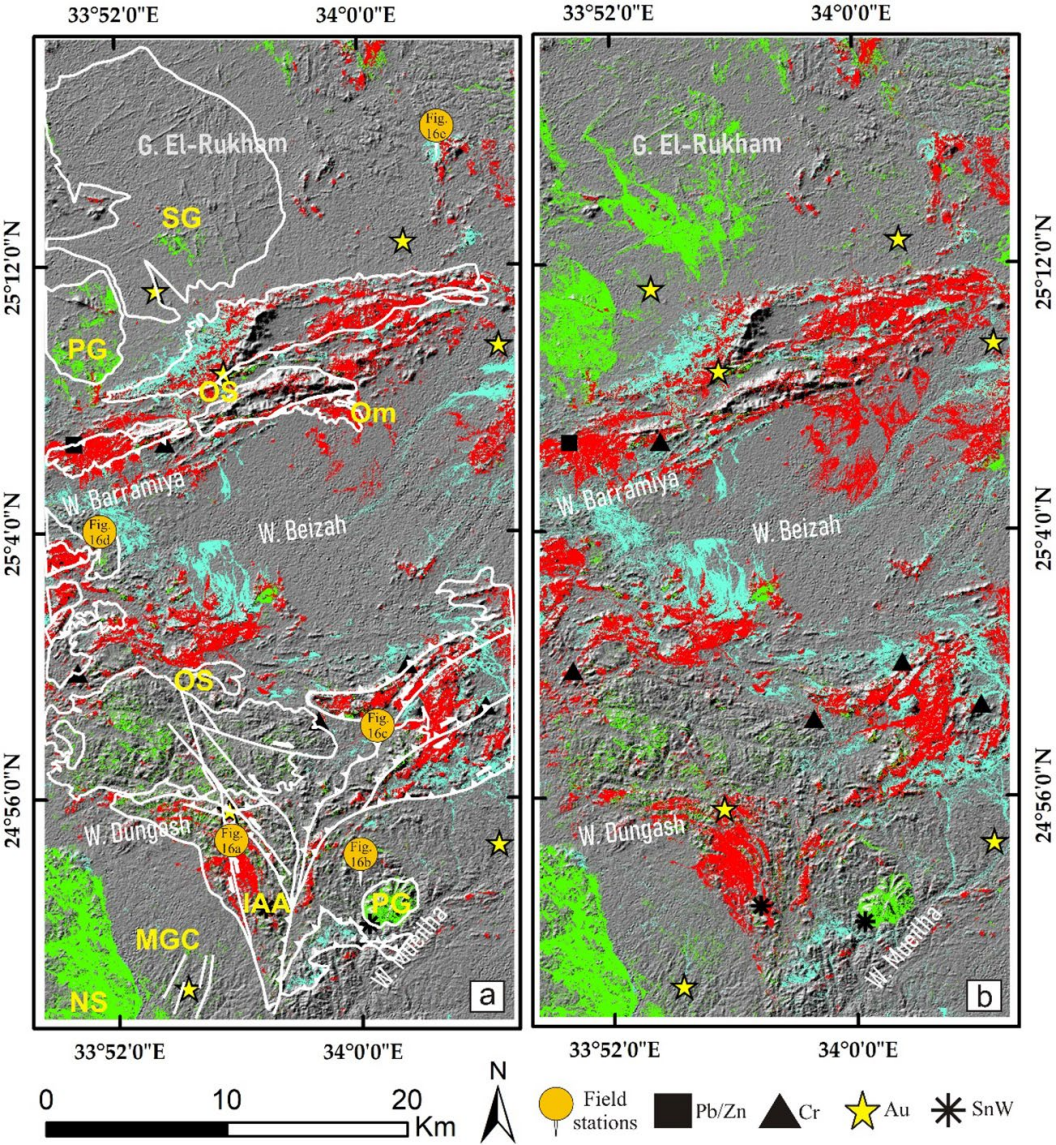
The areal distribution of rocks enriched with hydroxyl-bearing minerals (red), iron oxides (green), and ferrous oxides (cyan) derived utilizing (**a**) the four sensors, and (**b**) ASTER and sentinel 2. A great similarity is noticed revealing the potency and reliability of applying the combined sensor results in confirming hydrothermal alteration (see the location of the mineralized spots within or in between the detected altered zones). Also, the locations of each field photo (displayed in Fig. 16, i.e., a, b, etc.) are given over (**a**) (in yellow circles) highlighting the consistency of the detected alteration zones with our field investigations.

**Figure 16:**
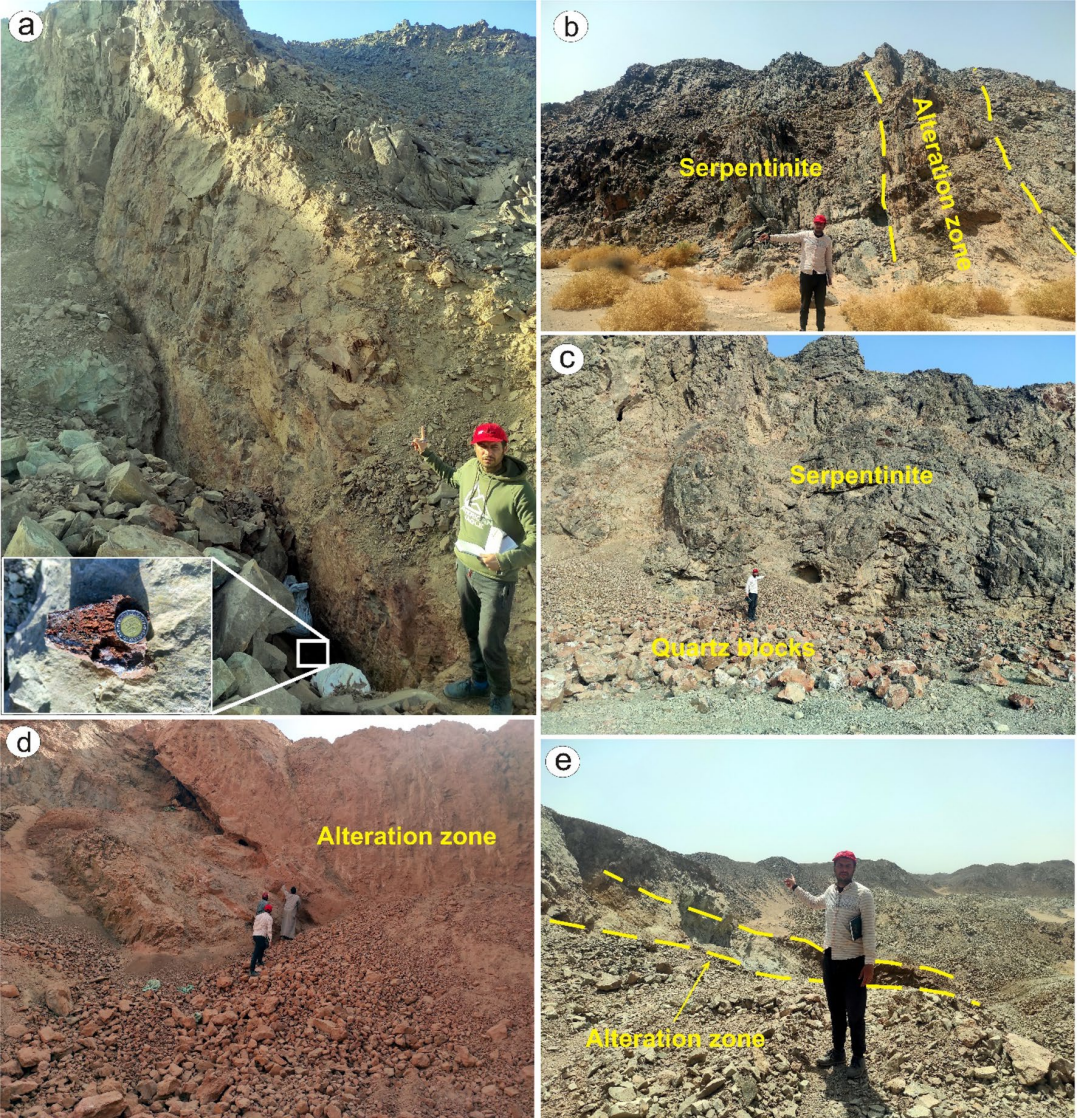
Field photos showing shear-related hydrothermal alterations of (**a**) Serpentinites exhibiting greenish field tint enclosing excavated gold mineralized quartz vein by random (artesian) miners. Note the reddish ferrous tarnish on the contact surface between the quartz and the host serpentinites. (**b**) Talc-carbonate zone with a pale whitish buff color associated with the darker serpentinites. (**c**) Falling down blocks and scree from little quartz stock invading the serpentinites. (**d**) Huge argillic-ferric alteration zone, several tens of meters across, in serpentinites leading to brick red deposits that are mined by the random (artesian) miners with rather low 1–2 ppm gold according to their estimation. (**e**) Intensive random mining in the alluvial and highly fractured loose (greenish propylitic altered) serpentinites was done by the random (artesian) miners using the loaders and guided by the metal detectors. (All the participants identified in these field photographs are authors of the current research. These photos are our own and we agreed to publish them.)

The main finding of the current research is highlighting the higher capability of collaborative usage of ASTER and Sentinel 2 data for better delineation of hydrothermal alteration zones. Our results coincided with airborne magnetic and radiometric data investigations, known mineralized zones, and previous studies. Additionally, fieldwork was performed to verify the detected hydrothermal alteration zones detected by the four datasets (Figs. 12, 13, and 14), and displayed in Fig. 15a to manifest the power of blended ASTER and Sentinel 2 (Fig. 15b) in targeting real hydrothermal alterations’ zones within the study area. A great harmony between the detected alterations and our field observations was noticed (Figs. 15 and 16). Our field examination revealed different types of hydrothermal alteration minerals or already mined zones, manifesting the role of current research in deciphering the hydrothermal alteration pattern of the study area.

According to the geological setting (Fig. 1), El Rukham- Mueilha district is occupied by various lithologies, including suprastructural Neoproterozoic Pan-African nappe assemblage (dismembered masses of ophiolitic mélange and island arc association) and syn to post-orogenic intrusive rocks which represents the main geological units within the Barramiya-Mubarak sector^88^. These lithologies are heavily altered by the magmatic fluids in the name of hematization (Fig. 16a), serpentinization (Fig. 16b,c), argillic (Fig. 16d), and propylitic alterations (Fig. 16e). These various types of alterations are linked with magmatic activity accompanied by the formation of ophiolites and island arc association, which were synchronously emplaced by multi-phase magmatism, which allowed the transfer of hydrothermal fluids and, consequently, the leaching of parent rocks.

**Figure 17:**
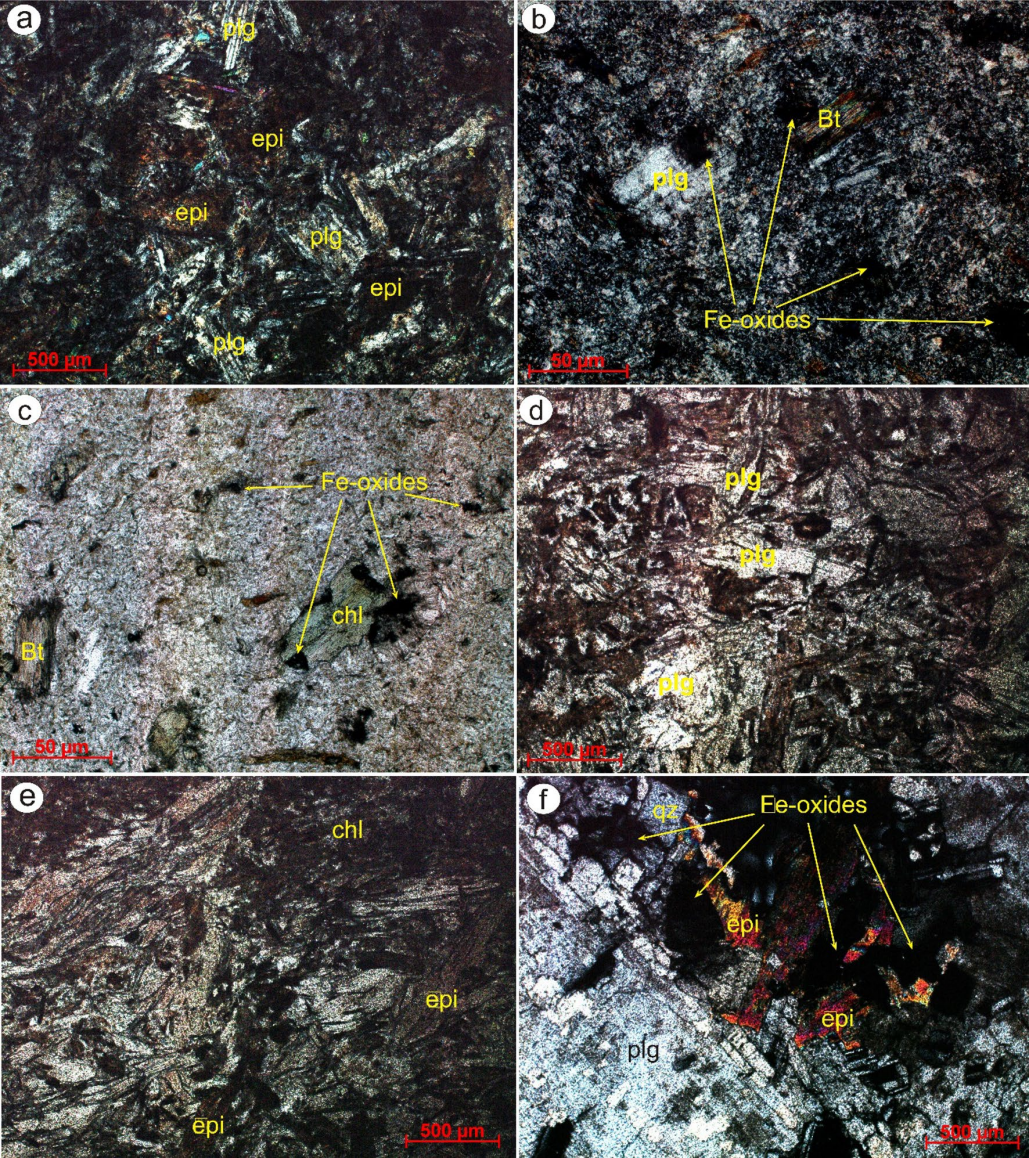
Thin-section photomicrographs showing the effect of hydrothermal alterations (**a**) within plagioclase (sericitized) and epidote (xN), (**b**) altered biotite and plagioclase phenocrysts (xN), and (**c**) iron oxides with altered biotite and chlorite phenocrysts (ppl) in altered metavolcanics exhibiting porphyritic texture. Depiction of typically-related hydrothermal alteration minerals including (**d**) altered plagioclase (ppl), (**e**) chlorite (xN), and (**e**, **f**) epidote (xN) within highly deformed lithologies. *Qz* quartz, *Pl* plagioclase, *Bt* biotite, *Chl* chlorite, *Epi* epidote.

The nappe assemblage is the most prevalent lithology and is made up of serpentinites (Fig. 16), a metagabbro-diorite complex, and metavolcanics embedded within a matrix of low-grade metasedimentary rocks. Serpentinite represents the low-temperature metamorphic/hydrous alteration product of igneous ultramafic rocks, while the metavolcanics are mainly andesitic in composition. This greatly matches our results that highlighted serpentinite rocks within the study area as hydroxyl-bearing alteration zones represented mainly by Gabal Um salim and Gabal Um salatit (Fig. 15). The island arc metavolcanics are mainly made of andesite and are affected by hydrothermal alterations, especially in the southwestern part of the study area around wadi Dungash (Fig. 15). Petrographically phenocrysts of heavily altered plagioclase and hornblende that have completely altered into epidote, iron oxides, or chlorite and are embedded in a fine-grained groundmass of plagioclase, hornblende, quartz, and mica, giving it a porphyritic texture (Fig. 17a–e).

This sequence is later intruded by multi-stage magmatism, including syn-orogenic and post-orogenic granites. These syn-orogenic granitoids are mostly exposed in Gabal El-Rukham, as opposed to the post-orogenic granite that is intermittently encountered in the research region (i.e., Mueilha granite). The syn-orogenic granites are dioritic in composition, including plagioclase, hornblende, quartz, and epidote, which represent with iron oxides the alteration product of hornblende (Fig. 17d,f).

## Discussion

Based on the previous hydrothermal alteration inspection using multi-sensor remote sensing data and airborne geophysical data, the main structural context of the study area, and previous studies^10,24,49,50,89^, the whole alteration pattern resulting from the four adopted datasets highlights their eligibility for detecting lithological units enriched with different types of hydrothermal alterations and their associated mineral deposits. After checking the eligibility of our results and coinciding with the main target of the current research, picking out the best sensor/s (based on their ability to delineate real alteration zones) to save time and effort, narrow the zones to be further exploited, and recommend them for further geological explorations is a critical task. The current research applied a quantitative areal (by measuring the areas of each hydrothermal alteration results and their intersections) overlay analysis (Table 3) of the sensor results to select the best results. The study strongly recommends blending at least two optical sensors for confirming the altered zones and narrowing the area to be further investigated. For example, hydroxyl-bearing minerals are disclosed in the range of 2.10–2.28 μm. If this range is covered by different wavelengths and bandwidths (e.g., Landsat 8 b7 187 nm, Sentinel 2 b12 180 nm, Sentinel 2 b12 180 nm), it is far better to confirm the presence of these minerals. This is clearly shown by the results in Fig. 12, where the overestimated area is larger than the confirmed pixels (resulting from the intersection of the four resultant patterns of the utilized sensors). Areal differences in the estimated zones directly result from changes in band designations and specifications for each sensor (e.g., the covered wavelength and bandwidth). Even for the same sensor, multi-temporal data could result in various anomalies^7^, thus a combination of two optical sensors is strongly recommended for further geological investigations to obtain focused real anomalies. It should be emphasized that the overestimated areas may have real altered pixels. However, the presence of false alteration zones has a higher probability compared to zones confirmed by the four datasets (altered pixels are often over or among the mineralized spots compared to overestimated areas, which have many distal pixels as shown in Figs. 12, 13, and 14). Thus, starting field investigations with these narrow zones is recommended to save the cost of further geophysical and geochemical analysis. Moreover, these zones could guide us to some parts of the overestimated areas through structural elements or any other evidence during field investigations.

In the current research, ASTER data potentiality in alteration mapping is unparalleled with the other three sensors. Without a doubt, ASTER decommissioning from 2009 has a negative effect on the geological remote sensing community. As a solution for confirming ASTER data anomalies and selecting another appropriate sensor to be combined with ASTER in further investigations, anomaly overlay analysis of the other 3 datasets was executed with ASTER data. It should be emphasized that the similarity between ASTER and Sentinel 2 results is eye-striking (Figs. 3 and 5) and strongly supports the geologic model of the hydrothermal mineral deposits distributed within the study area, meaning that Sentinel 2 data could be applied in performing a vigorous geological mapping. Additional spatial investigations of the results with the approximate locations of the mineral deposits (Fig. 15) corroborated the efficacy of fusing Sentinel 2 data (that has shown its efficacy in highlighting comparable zones affected by hydrothermal alteration) with ASTER data using an anomaly-overlaying selection technique. In this way, Sentinel 2 data could revive ASTER data and help solve the time gap issue that often arises with ASTER’s current investigations and reopen the door for applying the powerful SWIR spectral ranges in geological explorations. Moreover, magnetic and radiometric data implications clearly demonstrated how well the detected alteration anomalies of ASTER and Sentinel 2 are structurally controlled and mostly coincided with higher magnetic and K/eTh ratio anomalies.

It should be emphasized that the four datasets are eligible for comprehensive lithological and hydrothermal alteration mapping. However, In the current research, the favorability of ASTER and Sentinel 2 over L8 and ALI was assigned based mainly on the reality of the detected hydrothermal alteration zones (after comparison with airborne geophysical data and field investigations) which were attributed to the preferences of spatial and spectral resolutions as highlighted in Table 1. The latter highlighted that sentinel 2 data has 10 spectral ranges with a fine spatial resolution (10–20 m), and ASTER has 9 spectral bands with detailed SWIR ranges compared to Landsat 8 which has 8 spectral bands with a coarser pixel size of 30 m besides a single panchromatic band (15 m).

## Conclusions

The current research integrated multi-sensor remote sensing data, airborne magnetic and radiometric data, field observations, and petrographical examinations to decipher the hydrothermal alteration pattern of the study area, highlighting the efficiency of these multispectral datasets, and recommending the best for the geological community. Cross-linking results of ASTER, Sentinel 2, Landsat 8, Advanced Land Imager, magnetic, and radiometric data in geological and hydrothermal alteration mapping revealed the following.

1. The four datasets are eligible for comprehensive lithological and hydrothermal alteration mapping with acceptable areal differences attributed mainly to the powerfulness of the utilized wavelength and the desired target.
2. By comparing the results of at least two sensors, real alteration anomalies could be confirmed. This could narrow the area that may need to be investigated further with expensive geophysical and geochemical tests. Fieldwork should be commenced in these highlighted areas, which could lead to other anomalies that have not been found yet through other means, such as structural elements.
3. For a more detailed spectral analysis, ASTER data seems to be the best choice among the utilized sensors; however, a spatial analysis of the current study strongly recommends blending Sentinel 2 data results with ASTER findings to, confirm the altered zones, and narrow the area to be surveyed.
4. Band ratios, DPCA, and CEM techniques are extremely useful for deciphering the alteration patterns from the implemented datasets. Their results were pretty close to matching the structure of the area they looked at and how the mineralized spots were spread out. The current research strongly recommends integrating Sentinel 2 and ASTER data for deciphering further hydrothermal alteration patterns. Additionally, the placer deposits within the study area are of great interest due to the dominance of several alteration minerals in them.
5. Using the recommended integrated Sentinel 2 and ASTER data, the alteration zones were found to be in line with magnetic and radiometrically changed areas (with high magnetic and high K/eTh ratio anomalies) that are related to metalliferous minerals (like magnetite) and are located along the defined shear zone in the study area. Depth estimation analysis revealed that most of the anomalies have shallow sources (0–600 m); however, deep (up to 1500 m) structurally related anomalies also exist.
6. The present investigation unravels the hydrothermal alteration patterns (dominated mainly by OH-bearing minerals and iron oxides) and structural framework of the surveyed region by utilizing diverse data sources and scales, including satellite remote sensing images, airborne geophysical data, fieldwork, and petrographic examinations. Our research could enhance the likelihood of identifying genuine alteration zones for subsequent feasibility studies and exploitation.

## Data Availability

The datasets used and/or analyzed during the current study are available from the corresponding author upon reasonable request.
